# A New *Micromonospora* Strain with Antibiotic Activity Isolated from the Microbiome of a Mid-Atlantic Deep-Sea Sponge

**DOI:** 10.3390/md19020105

**Published:** 2021-02-11

**Authors:** Catherine R. Back, Henry L. Stennett, Sam E. Williams, Luoyi Wang, Jorge Ojeda Gomez, Omar M. Abdulle, Thomas Duffy, Christopher Neal, Judith Mantell, Mark A. Jepson, Katharine R. Hendry, David Powell, James E. M. Stach, Angela E. Essex-Lopresti, Christine L. Willis, Paul Curnow, Paul R. Race

**Affiliations:** 1 School of Biochemistry, University of Bristol, University Walk, Bristol BS8 1TD, UK; henry.stennett@bristol.ac.uk (H.L.S.); samuel.williams@bristol.ac.uk (S.E.W.); jorge.ojedagomez@bristol.ac.uk (J.O.G.); p.curnow@bristol.ac.uk (P.C.); 2 School of Chemistry, University of Bristol, Cantock’s Close, Bristol BS8 1TS, UK; luoyi.wang@bristol.ac.uk (L.W.); Chris.willis@bristol.ac.uk (C.L.W.); 3 Summit Therapeutics, Merrifield Centre, Rosemary Lane, Cambridge CB1 3LQ, UK; omar.abdulle@summitplc.com (O.M.A.); thomas.duffy@summitplc.com (T.D.); david.powell@summitplc.com (D.P.); 4 Woolfson Bioimaging Facility, University of Bristol, University Walk, Bristol BS8 1TD, UK; chris.neal@bristol.ac.uk (C.N.); j.mantell@bristol.ac.uk (J.M.); m.a.jepson@bristol.ac.uk (M.A.J.); 5 School of Earth Sciences, University of Bristol, Wills Memorial Building, Queens Road, Bristol BS8 1RJ, UK; k.hendry@bristol.ac.uk; 6 School of Natural and Environmental Sciences, Newcastle University, King’s Road, Newcastle upon Tyne NE1 7RU, UK; jem.stach@newcastle.ac.uk; 7 Defence Science and Technology Laboratory, Porton Down, Salisbury, Wiltshire SP4 0JQ, UK; aeelopresti@mail.dstl.gov.uk

**Keywords:** antibiotic, deep sea, sea sponge, bioprospecting, natural product, secondary metabolites, biosynthetic gene clusters, genome mining, nanopore sequencing

## Abstract

To tackle the growing problem of antibiotic resistance, it is essential to identify new bioactive compounds that are effective against resistant microbes and safe to use. Natural products and their derivatives are, and will continue to be, an important source of these molecules. Sea sponges harbour a diverse microbiome that co-exists with the sponge, and these bacterial communities produce a rich array of bioactive metabolites for protection and resource competition. For these reasons, the sponge microbiota constitutes a potential source of clinically relevant natural products. To date, efforts in bioprospecting for these compounds have focused predominantly on sponge specimens isolated from shallow water, with much still to be learned about samples from the deep sea. Here we report the isolation of a new *Micromonospora* strain, designated 28ISP2-46^T^, recovered from the microbiome of a mid-Atlantic deep-sea sponge. Whole-genome sequencing reveals the capacity of this bacterium to produce a diverse array of natural products, including kosinostatin and isoquinocycline B, which exhibit both antibiotic and antitumour properties. Both compounds were isolated from 28ISP2-46^T^ fermentation broths and were found to be effective against a plethora of multidrug-resistant clinical isolates. This study suggests that the marine production of isoquinocyclines may be more widespread than previously supposed and demonstrates the value of targeting the deep-sea sponge microbiome as a source of novel microbial life with exploitable biosynthetic potential.

## 1. Introduction

Antibiotic resistance is emerging as a global crisis, with a growing number of bacterial infections becoming harder to treat [1]. There has been no new, clinically viable class of antibiotic discovered since the 1980s [2]. According to the US Centre for Disease Control, we are already in a post-antibiotic era: common infections and minor injuries are becoming deadly, and routine medical procedures impossible to perform [3]. To tackle the emerging problem of antibiotic resistance, the identification of new antibiotics that are safe and effective against resistant microbes is of paramount importance [1]. 

Microbial natural products and their derivatives have, and will continue to be, an important source of medically important compounds. Recent advances in molecular, genetic and analytical methods are now enabling the rapid identification and characterisation of new natural product antimicrobials from microorganisms, driving a renaissance in the field [4,5]. One facet of this discovery effort is the exploitation of unsampled and extreme ecological niches. This has led to a particular interest in the deep sea, meaning ocean depths below ~1000 m, which are some of the least explored and most hostile environments on Earth. Ambient conditions in the deep sea can include low temperature, light, and oxygen content as well as high pressure and saline concentration. Microorganisms adapt to these extreme environments through a variety of metabolic innovations. For this reason, marine extremophiles are widely considered to be an important target for the discovery of novel natural products.

One potential source of deep-sea natural products are the abundant and diverse symbiotic microbial communities harboured by the sea sponges (phylum *Porifera*). This microbiome can account for up to 40% of the mass of a sponge [6,7] and commonly includes representatives of the Actinobacteria, which are famously versatile producers of secondary metabolites. The discovery potential is illustrated by the fact that over 30% of the ~15,000 unique marine natural products described to date have been isolated from sponge samples [8]. However, this has largely been confined to specimens from shallow waters and deep-sea sponges have been less well explored [9].

In recent studies we employed a remotely operated vehicle (ROV), deployed at depths of 0.5–4 km to recover sponge samples from previously unexplored regions of the mid-Atlantic seabed [10]. A subset of the microbiota of the sponges recovered during this expedition have been isolated and screened for their ability to biosynthesise natural products with antimicrobial activities. A number of the microorganisms recovered have been found capable of biosynthesising novel antimicrobial natural products [10]. Here we report the identification and characterisation of one such strain, which we term 28ISP2-46^T^, isolated from a sponge found 971 m below sea level. 16S rRNA gene and whole-genome sequencing of strain 28ISP2-46^T^ was consistent with its classification as a novel species of the genus *Micromonospora*, for which the name *Micromonospora robiginosa* sp. nov. (NCIMB number: 15402^T^, DSMZ number: 111791^T^) is proposed. Interrogation of the genome sequence identified a plethora of natural product biosynthetic gene clusters, many of which appear, based on bioinformatic analyses, to incorporate unusual biochemical features. The antitumour antibiotics kosinostatin and isoquinocycline B, which both comprise an anthracycline-like aglycon core coupled to a functionalised pyrrolopyrrole ring [11], could be readily isolated from 28ISP2-46^T^ fermentation broths. These compounds are shown herein to possess antibiotic activity against a variety of pathogenic bacteria, including Gram-positive, Gram-negative and multidrug-resistant strains, along with tumour cell line cytotoxicity. This study serves to further highlight the importance of the deep-sea sponge microbiome as a source of novel microbial life and natural product chemistry.

## 2. Results

### 2.1. Sponge Sample Collection and Processing

**Figure 1:**
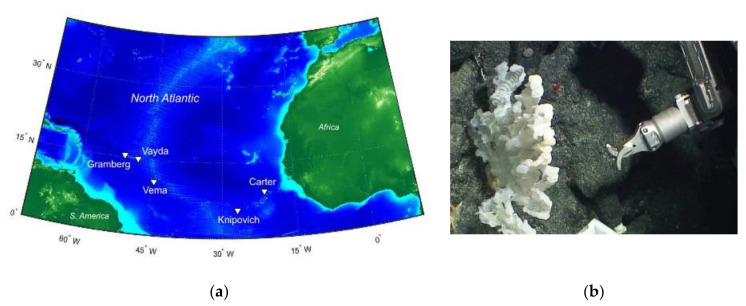
(**a**) Map of the sampling areas (arrowed) from the JC094 research cruise in the mid-Atlantic Ocean (created using ETOPO1 bathymetry). (**b**) The robotic arm of the remotely operated vehicle (ROV) used for collecting samples and one of the samples collected.

The Tracing Ocean Processes Using Corals and Sediments (TROPICS) research cruise (RRS James Cook expedition JC094) collected samples of *Porifera* (sea sponge) from five different sites across the equatorial Atlantic (Figure 1a). Sampling was executed between 0.5 and 4 km below the surface using a remotely operated vehicle (ROV) (Figure 1b) [10]. Once at the surface, samples were cryopreserved and transported to Bristol. 

Microbial culturing studies were performed using a demosponge sample (Supplementary Figure S1) collected at a sampling site in the eastern basin of the Atlantic around the Knipovich Seamount. Bacterial cultures isolated from the sponge sample were screened for antimicrobial activity [10], and bacteria able to inhibit the growth of any of the test strains were then taken forward for compound extraction and identification.

### 2.2. Isolation of a Micromonospora Strain with Antibiotic Activity

**Figure 2:**
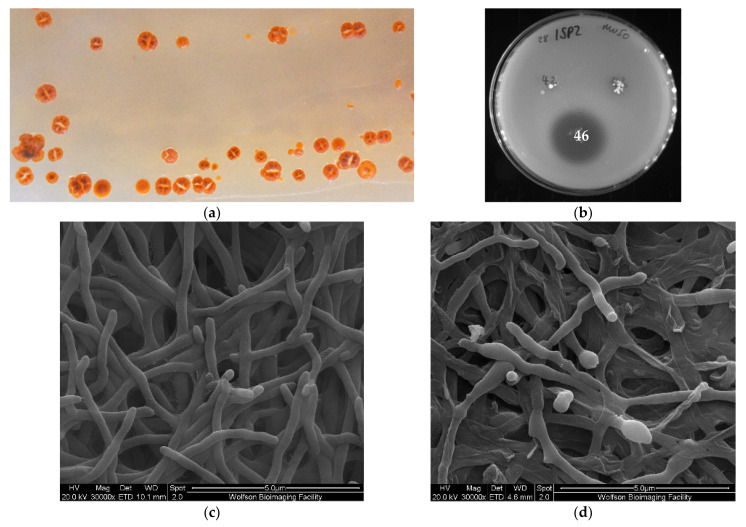
(**a**) Colonies of strain 28ISP2-46^T^ grown on ISP2 agar for 3 weeks. (**b**) Antibiotic activity of 28ISP2-46^T^ numbered ‘46’ against *Staphylococcus aureus* Mu50 in a soft agar overlay assay. (**c**) Scanning electron micrograph of cells of strain 28ISP2-46^T^ after growth on LB agar for 7 days at 28 °C. (**d**) Scanning electron micrograph of cells and spores (indicated by arrows) of strain 28ISP2-46^T^ after growth on LB agar for 3 weeks at 28 °C.

Culture-based screening with an agar overlay assay identified a deep-sea bacterial strain with antibiotic activity against multidrug-resistant Gram-positive bacteria such as *Staphylococcus aureus* Mu50 (methicillin and vancomycin resistant) (Figure 2b), as well as the Gram-negative pathogen *Acinetobacter baumannii* ATCC 19606. The strain was recovered from ISP2 (+ASW) agar incubated at 28 °C after 10 weeks post-inoculation and subsequently grew to confluence on ISP2 agar or in broth within 3 weeks. In lieu of detailed taxonomic assignment, we initially designated this strain 28ISP2-46^T^ according to our own arbitrary numbering system, which indicates that the strain was isolated at 28 °C and was the 46th colony to be isolated on ISP2 media. Visual inspection of colonies of 28ISP2-46^T^ on ISP2 agar revealed hard, wrinkled, rust-red colonies, turning to dark red/black upon sporulation, with a ‘molar tooth’ appearance (Figure 2a). The presence of a slight, pink, diffusible pigment was also observed. The strain is aerobic, Gram positive, non-motile and mesophilic. Scanning electron microscopy (SEM) indicated that the substrate hyphae are extensively branched and fragmented irregularly into rod-shaped, non-motile elements (Figure 2c). After a longer growth period (3 weeks), the strain visually began sporulating (dark red/black), and the SEM revealed these spores to be ovoid to spherical in shape, slightly rough on their surface and approximately 1 µm in diameter once fully grown (Figure 2d). 16S rRNA gene sequencing identified the species as belonging to the genus *Micromonospora* (99%; accession number MW003705.1), within the class Actinobacteria (Tables S2 and S3).

### 2.3. Identification of Quinocycline Antibiotics

In an effort to establish the identity of the molecule(s) responsible for the observed antimicrobial activity, 28ISP2-46^T^ was cultured in 10 mL ISP2 broth for 3 weeks. Cells were harvested by centrifugation, and the supernatant and pellet fractions extracted in ethyl acetate. Supernatant fractions exhibited antibacterial activity (Figure S2). There was no loss of activity in supernatant samples pretreated with Proteinase K, indicating that the active molecule(s) was not a peptide (Figure S2). 

**Figure 3:**
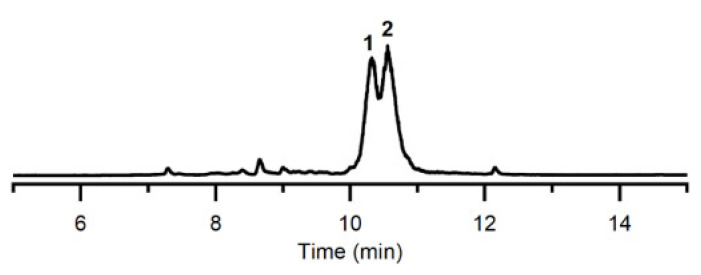
HPLC-ELSD chromatogram of the crude extract of *Micromonospora* sp. 28ISP2-46^T^.

**Figure 4:**
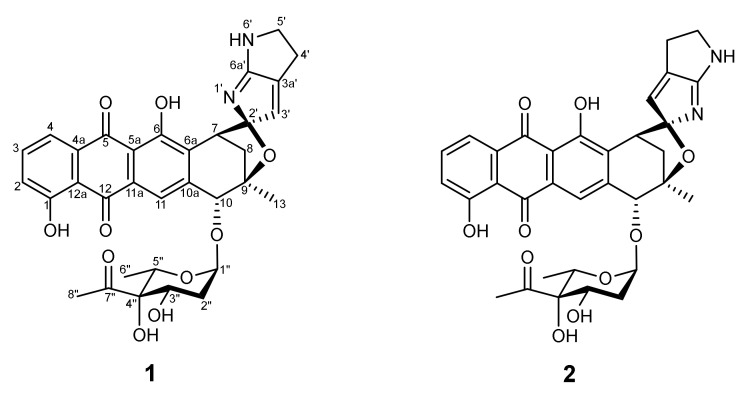
Structures of the two quinocycline compounds: quinocycline B (‘kosinostatin’) (**1**) and isoquinocycline B (**2**).

For the isolation and characterisation of metabolites, it proved necessary to scale up the fermentation of 28ISP2-46^T^ to 2 L of culture volume. It has been previously established that a higher yield of product can be obtained when the bacteria are grown in a less-rich media [11]. So, for these large-scale fermentations, 28ISP2-46^T^ was grown in 25% ISP2 with artificial sea water (ASW). The supernatant was extracted with ethyl acetate and evaporated in vacuo to give a crude extract, which was subjected to LC–MS analysis (Figure 3). The crude extract was further purified by HPLC to yield compounds **1** (~ 2 mg) and **2** (~3 mg). Compound **1** was unstable and isomerised to **2** at room temperature. HR-MS and NMR analysis identified compound **2** as isoquinocycline B (Figure 4, Supplementary Figures S3–S7), previously isolated and identified from *Streptomyces* [12,13,14,15]. This was further confirmed by comparing the NMR data of **2** with those of a standard sample of isoquinocycline B, provided by Prof. Gong-Li Tang (Shanghai Institute of Organic Chemistry, Chinese Academy of Sciences) (data not shown). Compound **1** was proposed to be kosinostatin, although full characterisation was not possible as it was unstable.

The two quinocycline antibiotics (**1**–**2**, Figure 4) are similar to anthracyclines and the overall structure is composed of an aglycon core, a sugar moiety and a pyrrolopyrrole ring (Figure 4) [12]. Compounds **1** and **2** have been shown to have antibiotic activity and to also inhibit DNA topoisomerase IIα [14]. 

### 2.4. Biological Activities

The minimum inhibitory concentration (MIC_90_) of the purified compound (**2**) was determined against *A. baumannii* ATCC 17978, *Pseudomonas aeruginosa* ATCC 33359, *Escherichia coli* BW25113, *Klebsiella pneumoniae* ATCC 10,031 and *Staphylococcus aureus* SH1000, along with genetically modified strains to help ascertain any mechanisms of resistance (Table 1). These GM strains included an efflux knockout (KO) strain of *A. baumannii* (CCD167h1.1), a derivative of ATCC 17,978 with deletion of two efflux pumps (Δ*adeIJ*, ΔA1S_3447-3446); an efflux KO of *P. aeruginosa* (CCD047), a derivative of NCTC 11451 with deletion of four efflux pumps (Δ*mexAB*, Δ*mexCD*, Δ*mexVW*, Δ*mexXY*); an efflux KO of *E. coli* (CCD121a8), a derivative of BW25113 with deletion of six efflux pumps (Δ*acrAB*, Δ*acrD*, Δ*emrAB*, Δ*macAB*, Δ*mdfA*, Δ*mdtK*); and *E. coli* CCD181-1 (BW25113 imp4213), an increased membrane permeability (IMP) strain (Table S1).

Isoquinocycline B (**2**) showed good antibiotic activity not only against the Gram-positive strain *S. aureus* SH1000, but also the Gram-negative strains *K. pneumoniae* ATCC 10031 and *A. baumannii* ATCC 17978. However, there was no activity against the other Gram-negative strains, except the efflux KO mutant strains, indicating that these strains are unable to transport the compound out of the cell, and thus demonstrating the mechanism of resistance of these strains.

The in vitro cytotoxicity of isoquinocycline B (**2**) was tested against the human liver cancer cell line HepG2 (Table 2). The compound exhibited relatively high activity against this cell line, with a half inhibitory concentration (IC_50_) of 13.3 µM and 0% cell viability at 100 µM.

### 2.5. Genome Sequencing and Assembly

Highly accurate short-read sequencing (Illumina MiSeq) was combined with less accurate long-read sequencing (Nanopore MinION) to produce a high-quality hybrid assembly of the genome of 28ISP2-46^T^. The Unicycler [16] software was used to produce a hybrid assembly of the 28ISP2-46^T^ genome (Table 3). This generated an Illumina-only assembly using the SPAdes assembler [17] and assessed the number of connections between contigs on the assembly graph. The less-accurate longer reads from Nanopore sequencing were used to resolve any repeat regions, eliminating all connections to produce a single contig of 6.64 Mb (Table 3). The average coverage was 290×, which was consistent across the assembly, and 98.5% of the Illumina reads mapped back to the assembly with an error rate of 0.44%. The complete genome sequence of strain 28ISP2-46^T^ has been deposited at DDBJ/ENA/GenBank under the accession number CP059322. The annotated genome features are listed in Supplementary Table S4 and the Clusters of Orthologous Groups analysis is presented in Supplementary Figure S9.

An *in silico* method was used to compare the genome of strain 28ISP2-46^T^ with those of other *Micromonospora* species. The Genome-to-Genome Distance Calculator (GGDC) [18] was employed to infer whole-genome distances which mimic DNA–DNA hybridisation (DDH), thus reporting DDH values and the probability that two bacteria belong to the same species. GGDC was used to align the genome assembly of strain 28ISP2-46^T^ against all 152 sequenced *Micromonospora* genomes on the NCBI database. The closest relatives are *Micromonospora humi* (DDH = 49.2, %GC difference = 0.12, probability same species = 17%) and *Micromonospora sediminicola* (DDH = 40.2, %GC difference = 0.09, probability same species = 3%). A DDH value below 70% indicates that two strains belong to different species, supporting the proposal that strain 28ISP2-46^T^ represents a novel species of the genus *Micromonospora*.

**Figure 5:**
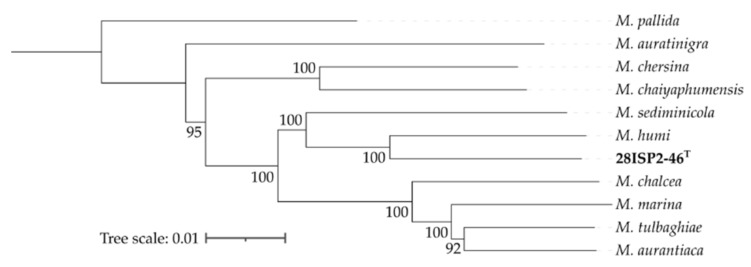
Phylogenetic tree for strain 28ISP2-46^T^ and the other group IA *Micromonospora* species generated using the TYGS and drawn with iTOL. The tree was constructed using FastME 2.1.6.1 [20] from GBDP distances calculated from genome sequences. The branch lengths are scaled in terms of GBDP distance formula d5. The numbers above branches are GBDP pseudo-bootstrap support values from 100 replications, with an average branch support of 98.4% and a delta statistic of 0.135. The tree was rooted using *Micromonospora pallida* as the outgroup.

The Type Strain Genome Server (TYGS) was used to generate a phylogenetic tree for strain 28ISP2-46^T^ (Figure 5), by using a whole-genome BLAST distance phylogeny approach [19].

The Antibiotics and Secondary Metabolites Analysis Shell (AntiSMASH) [21] was used to identify putative biosynthetic genes, organise them into predicted clusters, and find their closest known relatives to predict the natural products of the clusters within the genome of 28ISP2-46^T^. AntiSMASH identified 19 putative clusters in the draft genome including a candidate gene cluster for kosinostatin biosynthesis (Table 4). 

### 2.6. Quinocycline-Producing Gene Cluster

**Figure 6:**
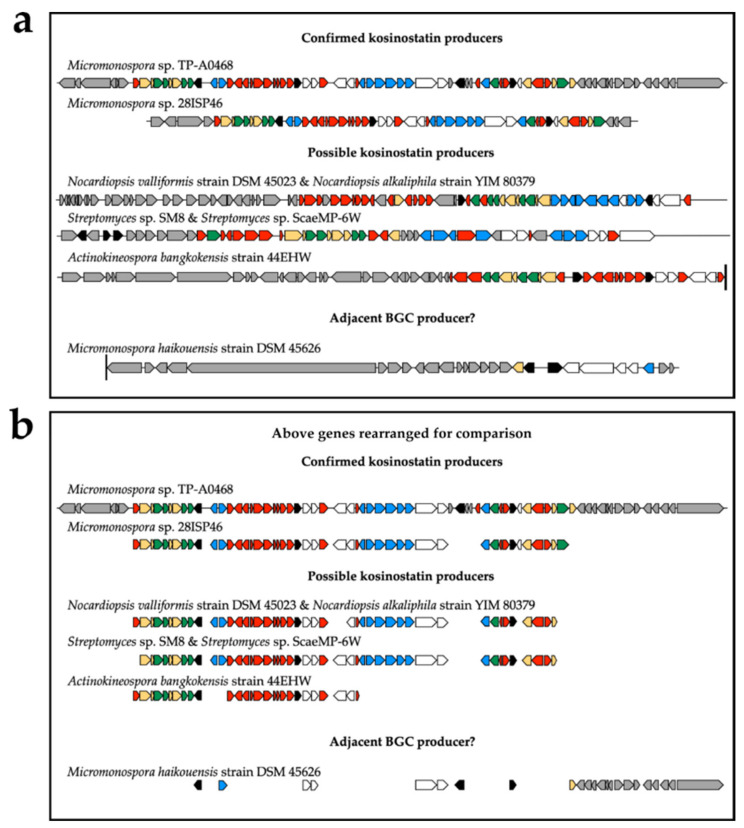
(**a**) ClusterBLAST [21] hits for the kosinostatin cluster. Homologues of *kst* genes are coloured as follows: *kstA* genes red, *kstB* genes yellow, *kstC* genes green, *kstD* genes blue, *kstRg* genes black, *kstRs* genes white, and genes uninvolved in kosinostatin biosynthesis grey. A black line at the end of a cluster indicates that it is close to the end of a contig. Gene sizes not to scale. The NCBI protein IDs for first and last coloured genes of each cluster are as follows: *Micromonospora* sp. TP-A0468 AFJ52719.1 and AFJ52701.1; *Micromonospora* sp. 28ISP2-46^T^ QLQ36639.1 and QLQ36600.1; *N. valliformis* WP_017579213.1 and WP_017579258.1; *N. alkaliphila* WP_017604198.1 and WP_017604153.1; *Streptomyces* sp. SM8 PKA38649.1 and PKA38729.1; *Streptomyces* sp. ScaeMP-6W SCE31237.1 and SCE31955.1; *A. bangkokensis* OLR89622.1 and OLR89598.1; *M. haikouensis* SCF11188.1 and SCF11278.1. (**b**) The same gene clusters rearranged for ease of comparison. Coloured as 6a.

The ‘kosinostatin’ biosynthetic gene cluster (BGC) reported for strain 28ISP2-46^T^ (Table 4) is only the second confirmed from a quinocycline-producing strain. The first was identified from *Micromonospora* sp. TP-A0468, which was shown to produce both **1**, quinocycline B (‘kosinostatin’), and **2**, isoquinocycline B, despite the irreversible isomerisation of **1** to **2** at room temperature [12,14]. The BGC from 28ISP2-46^T^ can be compared with that of *Micromonospora* sp. TP-A0468 [22] to better define the BGC boundaries. AntiSMASH defined the two BGCs as 77% similar based on gene conservation (Table 4) and as indicated in Figure 6, the two BGCs are closely related. Ma et al. defined eight groups of genes in the *Micromonospora* sp. TP-A0468 BGC, the predicted functions of which are shown in Table 5 [22].

Relative to the *Micromonospora* sp. TP-A0468 BGC, six ORFs are missing in strain 28ISP2-46^T^. There are no homologs of *kstU1, kstRg3, kstU2, kstU3, kstRg4*, or *kstA14*. KstU1 and KstU2 are hypothetical proteins with no putative conserved domains in the BLAST database. KstRg3 and KstRg4 were predicted to be transcription factors [22]. Therefore, the two producer strains may have evolved different regulatory mechanisms for the production of the quinocyclines, or these genes may not act on either ‘kosinostatin’ cluster. Ma et al. [22] identified ORF KstRg4 and included it in their ‘kosinostatin’ Minimum Information about a Biosynthetic Gene (MiBIG) cluster. However, this putative gene could not be found in the *Micromonospora* sp. TP-A0468 kosinostatin BGC sequence that was deposited in the NCBI database. KstA14 was annotated as a dehydrogenase by Ma et al. [22] because a weakly related gene from the zebrafish *Danio rerio* was annotated with this function. This BLAST search was repeated, but a related gene from *D. rerio* could not be found and all of the sequences returned were hypothetical proteins. Aligning the KstA14 deduced amino acid sequence with all of the translated ORFs in the genome of strain 28ISP2-46^T^ did not return any related ORFs. KstA14 does not have any putative conserved domains in the BLAST database, and because it is not conserved between the two clusters, it is unlikely to be involved in kosinostatin biosynthesis. Ma et al. predicted that KstA14 catalyses an auxiliary reaction in the biosynthesis of the kosinostatin anthracycline [22], but this reaction could be catalysed by another KstA protein.

The major difference between the two BGCs is the absence of *kstB7* in the strain 28ISP2-46^T^ cluster. Ma et al. used *kstB7* to define one boundary of the TP-A0468 BGC because a ∆*kstB7* strain had reduced kosinostatin production [22]. It was predicted that KstB7, which has homology to a trypsin-like serine protease, removes a formyl group from a pyrrole precursor (Figure S8). None of the putative peptidases near the strain 28ISP2-46^T^ *kst* BGC show significant similarity to *kstB7* and are all outside the cluster. Aligning the *kstB7* nucleotide sequence with the entire strain 28ISP2-46^T^ genome returns a homologue with 81% identity, but this gene is nearly 1 Mb removed from the kosinostatin cluster. Therefore, it seems unlikely that KstB7 is involved in kosinostatin biosynthesis, and the deformylation reaction may be catalysed by an enzyme outside of the BGC or happen spontaneously.

ClusterBLAST [21] was used to identify BGCs with similarity to the 28ISP2-46^T^ kosinostatin cluster (Figure 6a). The boundaries of the BGCs were identified by ClusterBLAST and the ORFs were aligned to those of the TP-A0468 cluster to find *kst* gene homologues. As indicated in Figure 6b, *Nocardiopsis valliformis, Nocardiopsis alkaliphila, Streptomyces* sp. SM8 and *Streptomyces* sp. ScaeMP-6W have almost complete kosinostatin BGCs. *Actinokineospora bangkokensis* may also have a complete kosinostatin BGC, although the cluster is interrupted by the end of a contig (Figure 6a). None of these strains have yet been reported to produce kosinostatin. It is notable that these species also lack the six genes missing from strain 28ISP2-46^T^, implying that these are not necessary for kosinostatin biosynthesis. The genome of *Micromonospora haikouensis* has homologues of all of the genes downstream of the TP-A0468 cluster as well as of *kstB7*, which suggests that *kstB7* is part of a nearby cluster that includes a polyketide synthase. It also has homologues of some *kstRs* and *kstRg* genes and *kstD2*, but no other *kst* homologues within twenty ORFs of the BGC. This could indicate that *Micromonospora haikouensis* once had a kosinostatin cluster which has since been deleted. 

### 2.7. Genome Comparison of Strain 28ISP2-46^T^ with Other Micromonospora Species

Carro *et al*. [23] classified the *Micromonospora* species into five groups based on their whole-genome sequences. *M. humi*, the closest relative of strain 28ISP2-46^T^, belongs to subgroup Ia, which also includes *M. aurantiaca, M. auratinigra, M. chalcea, M. chaiyaphumensis, M. chersina, M. marina, M. sediminicola*, and *M. tulbaghiae*. The genomes of these species were analysed using antiSMASH to identify BGCs unique to strain 28ISP2-46^T^ that may be of special interest for genome mining (Table S3). Some of the predicted BGCs from the genome of 28ISP2-46^T^ are present in all of the other strains in group Ia, but some are in only a few others and two are unique to strain 28ISP2-46^T^, including a cluster with 63% similarity to the feglymycin BGC and the kosinostatin BGC. These observations indicate that none of the most closely related strains to strain 28ISP2-46^T^ produce kosinostatin and it is unique within the group.

## 3. Discussion

In this study, we report the identification of a novel bacterial strain (28ISP2-46^T^) within the class *Actinobacteria* from a deep-sea sponge, harvested from a depth of 971 m in the mid-Atlantic Ocean. The strain was screened for antibiotic activity against a variety of pathogenic bacterial strains and was shown to have antibiotic activity on agar against predominantly Gram-positive species. Subsequently, two quinocycline compounds were isolated from the growth medium of this strain, which were identified as the known compounds quinocycline B (kosinostatin) (**1**) and the corresponding diastereoisomer isoquinocycline B (**2**) [12,13,14,15]. Based on the compounds identified and genome sequencing, strain 28ISP2-46^T^ is clearly distinct from other closely related type strains. Thus, it is proposed that strain 28ISP2-46^T^ is assigned to a novel species within the genus *Micromonospora*, for which the name *Micromonospora robiginosa* sp. nov. is proposed. The type strain is 28ISP2-46^T^.

Quinocycline antibiotics have been identified in a number of different *Streptomyces* strains over the past 60 years [12,13,14,15,24]. The structures of the compounds were eventually fully solved in 2002 by Igarashi et al. [12], revealing an unusual glycosylated anthraquinoid tetracycle with a pyrrolopyrrole ring spirally conjugated to the aglycon core. The same study [12] supports our own observations of the reported spontaneous isomerisation of quinocycline B (compound **1**) into isoquinocyclin B (compound **2**). Although attempts have been made to chemically synthesise the quinocyclines, only synthesis of the branched octose [25], pyrrolopyrrole substructure [26,27] and intermediate lactone [28] have so far been achieved. Thus, biosynthesis provides the only current route to these compounds and the insights gained here could facilitate strain engineering to enhance this production. 

Isoquinocycline B (**2**) exhibited antibiotic activity against Gram-positive strain *S. aureus* SH1000, as well as the Gram-negative strains *K. pneumoniae* ATCC 10,031 and *A. baumannii* ATCC 17978. Experiments using knockout strains suggested that resistance in other Gram-negative strains was mediated by efflux. Isoquinocycline B was also toxic to the HepG2 liver cancer cell line, in agreement with the anticancer properties of kosinostatin highlighted by Igarashi et al. [29]. The mechanism of cytotoxicity is indicated to be by inhibition of DNA topoisomerase [14], similar to other DNA intercalators such as the aromatic polyketides daunorubicin and doxorubicin, which have a similar anthracycline core [30].

It is increasingly possible to routinely assemble genomes that are highly contiguous and accurate [31]. This is desirable because a more contiguous genome is easier to mine for biosynthetic gene clusters. The hybrid assembly performed here using Nanopore MinION and Illumina MiSeq data generated an accurate genome of strain 28ISP2-46^T^ into a single contig. The coverage was high and consistent across the assembly. 

Strain 28ISP2-46^T^ has considerable potential for the production of natural products, with 19 putative secondary metabolite-producing gene clusters identified in the genome by AntiSMASH. One of the identified BGCs had a 77% similarity to the kosinostatin-producing biosynthetic gene cluster from *Micromonospora* sp. TPA0468 [22], indicating that it is the potential gene cluster used to produce the quinocycline molecules (**1** and **2**) purified from the growth media. It is promising that the other 18 biosynthetic gene clusters identified might also produce compounds either in low quantities compared to **1** and **2**, or under different conditions. Work will continue to mine these clusters, particularly those with low sequence similarity to known clusters, for novel, antimicrobial compounds. Recently, Zhou et al. (2019) [32] forced the production of a different secondary metabolite in *Micromonospora* sp. TP-A0468 by deletion of the kosinostatin-producing cluster. Cloning of the unknown gene clusters of strain 28ISP2-46^T^ into heterologous expression strains could also yield production of other secondary metabolites.

Analysis of the genome by the Genome-to-Genome Distance Calculator (GGDC) and the Type Strain Genome Server (TYGS) reveals that strain 28ISP2-46^T^ is a novel species of the genus *Micromonospora*. Despite there being no whole-genome sequence of *Micromonospora* sp. TP-A0468 published, the low similarity of the kosinosatin-producing gene cluster of TP-A0468 with the predicted cluster of the strain 28ISP2-46^T^ and visual comparison of bacterial colonies on the same type of agar [14] demonstrates these strains are not identical. The gene discrepancies between the BGCs of the two strains include the absence of seven predicted genes in the 28ISP2-46^T^ cluster, indicating that the previous cluster gene annotation of TP-A0468 was not accurate. In particular, the absence of the predicted gene for the trypsin-like serine protease KstB7 indicates that the predicted deformylation reaction of the kosinostatin precursor [22] (Figure S8) is likely catalysed by another, unknown enzyme or happens spontaneously. Comparison with other possible kosinostatin-producing BGCs from other strains supports the case that these genes are probably not involved in kosinostatin biosynthesis. These other strains of bacteria that have BGCs homologous to the kosinostatin-producing clusters from both *Micromonospora* sp. TP-A0468 and the strain 28ISP2-46^T^ (Figure 6) have not yet been shown to produce kosinostatin. This is despite research that has indicated that most of these strains do produce at least one form of anti-infective compound [33,34,35,36], demonstrating that the *kst* BGCs may be ‘silent’ and could be activated to produce the kosinostatin under the right conditions.

According to the closest relatives of strain 28ISP2-46^T^ it is part of the *Micromonospora* group Ia. The species within this group share a number of homologous BGCs. However, only 28ISP2-46^T^ carries BGCs that are homologous to those that produce kosinostatin and feglymicin. This indicates that the production of kosinostatin is unique to this strain within group Ia. Feglymicin is a 13mer peptide antibiotic originally isolated from *Streptomyces* sp. DSM 11,171 [37], which is an inhibitor of enzymes involved in peptidoglycan biosynthesis [38]. 28ISP2-46T has a BGC with a 63% similarity to the feglymicin BGC, showing that this strain may have the potential to produce this, or a similar, antibiotic compound.

In conclusion, here we report the identification and characterisation of a novel species of the genus *Micromonospora*, which possesses the capacity to biosynthesise a diverse array of natural products, including the anticancer antibiotics kosinostatin (**1**) and isoquinocycline B (**2**). Isoquinocycline B (**2**), the more stable isomer of kosinostatin (**1**), was shown herein to be active against a diverse panel of pathogenic bacteria, including multidrug-resistant clinical isolates. This study can be considered a proof of concept, demonstrating the value of bioprospecting approaches that target deep-sea sponge microbiota. We propose that microorganisms from the deep sea represent an underexploited resource in the quest to identify new natural product based antimicrobial lead compounds.

### Description of ‘Micromonospora robiginosa’ sp. nov.

The novel strain 28ISP2-46^T^ will be hereafter known as *Micromonospora robiginosa* sp. nov.

*Micromonospora robiginosa* sp. nov. (ro.bi.gi.no’sa. L. fem. adj. *robiginosa*, referring to the colour of iron rust, dark red, from the colour of the bacterial colonies growing on ISP2 agar). 

*M. robiginosa* sp. nov. is an aerobic, Gram-positive, non-motile, mesophilic actinomycete that forms hard, rust-red-coloured colonies on ISP2 agar. It produces a peach-pink diffusible pigment, particularly visible on pale-coloured media. Substrate hyphae are extensively branched and fragmented irregularly into rod-shaped, non-motile elements. It produces spores that are ovoid to spherical, slightly rough, non-motile, and approximately 1 µm in diameter once fully grown. Growth of the strain is between 20 and 37 °C, between pH 5 and 9, and in the presence of up to 4% NaCl on unbuffered Luria–Bertani agar. The strain grows optimally at 28 °C, at pH 7, and in the presence of 0% NaCl. The genome size of the type strain is 6.64 Mb and its DNA G+C content is 72.5 mol %. The strain was isolated from a deep-sea sponge from the Knipovich Seamount in the Atlantic Ocean. The type strain is 28ISP2-46^T^ (NCIMB number: 15402^T^, DSMZ number: 111791^T^).

## 4. Materials and Methods

### 4.1. Collection of Sponge Samples

Sponge samples were collected as part of a research cruise in the equatorial Atlantic; the Tracing Ocean Processes Using Corals and Sediments (TROPICS) expedition JC094, (13 October 2013–30 November 2013). Five locations were selected for sampling: from east to west, the Carter and Knipovich seamounts in the eastern basin, the Vema fracture zone at the Mid-Atlantic Ridge and the Vayda and Gramberg seamounts in the western basin. The sponges were subsampled in a controlled-temperature (4 °C) laboratory on board the ship before flash freezing them for storage at −80 °C. The sponge used for the current study was collected at a depth of 971 m, from the Knipovich seamount in the eastern basin of the Atlantic Ocean (5° 37.5038′ N, 26° 57.4780′ W). It was taxonomically assigned to the Class Demospongiae by microscopic identification of sigma-C microscleres during the research cruise (Supplementary Figure S1).

### 4.2. Sponge Processing and Isolation of Bacterial Strains

The sponge sample was prepared and homogenised as described by this group in Williams et al. 2020 [10]. The sponge homogenate was serially diluted (10^−1^ to 10^−4^) with sterile artificial sea water (ASW; Crystal Sea Marine Mix, Marine Enterprise International, made to the manufacturer’s instructions) and spread onto a variety of agar types [10], in duplicate. All agar was made with ASW and supplemented with cycloheximide (10 µg/mL) to inhibit fungal growth. To maximise recovery of environmental isolates, media included sodium pyruvate (100 µg/mL) [39], trace metal solution (Trace Metal A5 with co, Sigma-Aldrich; 1 mL/L) and Basal Medium Eagle concentrated solution (BME, Sigma Aldrich; 1 mL/L) [40]. Nalidixic acid (30 mg/mL) was also added to some media to inhibit the growth of Gram-negative bacteria. Duplicate plates were incubated at 4 and 28 °C for 4–10 weeks. A large number of colonies with a wide variety of morphologies were picked and streaked onto fresh agar of the same type to grow as a monoculture in the same isolation temperature. The axenic strains were then directly stocked from the agar plate into Microorganism Preservation System Protect Cryotubes (Technical Service Consultants Ltd., Heywood, UK) and stored at −70 °C.

### 4.3. 16S rRNA Gene Sequencing of Strain 28ISP2-46^T^

Genomic DNA from strain 28ISP2-46^T^ was purified with a GenElute Bacterial Genomic DNA Kit (Sigma) following the manufacturer’s protocol. A sample of the resulting DNA solution (1 µL) was mixed with 12.5 µL of CloneAmp HiFi PCR premix (ClonTech), 9.5 µL of ddH_2_O, and 1 µL each of the primers 8F (AGAGTTTGATCCTGGCTCAG) and rP2 (ACGGCTACCTTGTTACGACTT) at 10 µM. The following thermocycler program was used: initial denaturation (1 cycle): 10 min at 95 °C. Amplification (35 cycles): 1 min at 95 °C, 1 min at 58 °C, 1.5 min at 72 °C. Final extension (1 cycle): 10 min at 72 °C. For negative controls, ddH_2_O was used. The presence of single bands was confirmed using gel electrophoresis and PCR products were purified with a QIAquick PCR cleanup kit (Qiagen). Sequencing (Eurofins Genomics) using the 8F and rP2 primers was performed to identify the strains.

### 4.4. Cultivation Conditions of 28ISP2-46^T^

The ability of strain 28ISP2-46^T^ to grow under a range of conditions was investigated by culturing the strain on a range of Luria–Bertani (LB) agar with differing compositions. The pH of unbuffered LB agar was adjusted between 5 and 12 at increments of 1 pH unit, and the NaCl content prepared between 0 and 12% *w/v* at 2% increments. The strain was incubated on these media at 28 °C. The strain was also incubated on standard LB agar at 4, 15, 20, 28, 34, 37, and 45 °C. It was additionally grown anaerobically on standard LB agar at 28 °C in candle jars with AnaeroGen 2.5 L bags (Thermo Scientific). In all cases, the cultures were incubated for four weeks and each test was repeated in triplicate.

### 4.5. Scanning Electron Microscopy of 28ISP2-46^T^

The strain 28ISP2-46^T^ was cultured on sterile 0.2 µm cellulose acetate filters (Sartorius Stedim Biotech) on LB agar (0% NaCl) for 1 week, and for an additional 2 weeks for spore identification. The filter paper was then peeled off and the specimens prepared for scanning electron microscopy (SEM). The filters were washed for 10–20 s in 0.1 M cacodylate buffer prior to fixing in 2.5% glutaraldehyde in 0.1 M cacodylate buffer (pH 7.3) for 1 h. They were then washed in cacodylate buffer and fixed in 1% osmium tetroxide in 0.1 M cacodylate buffer for 30 min. After buffer and dH_2_O washes, the membranes were dehydrated with increasing ethanol concentrations and loaded in 100% ethanol onto the critical point dryer (Leica EM CPD300) to dry. They were then sputter coated with Au/Pd using an Emitech K575X coating unit and imaged in a Quanta 200 Field Emission SEM (FEI, Thermo Fisher, Hillsboro, OR, USA). 

### 4.6. Soft Agar Overlay Antibiotic Activity Assay

Strain 28ISP2-46^T^ was inoculated as a small circle onto ISP2 agar (0.4% yeast extract, 1% malt extract, 0.4% dextrose, 3.3% ASW, 1.5% agar) and incubated at 28 °C for 15 days. Bacterial strains *Staphylococcus aureus* Newman, *S. aureus* Mu50, *Pseudomonas aeruginosa* PA01, *Escherichia coli* BW55113, *Klebsiella pneumoniae* NCTC 5055 and *Acinetobacter baumannii* ATCC 19,606 were grown in Mueller–Hinton broth (Sigma), at 37 °C, for 16 h. Each culture was suspended at OD_600_ = 0.01 in warm (42 °C), soft Mueller–Hinton agar (0.75% agar) and 10 mL of this overlay agar poured onto the circle of 28ISP2-46^T^. Plates were incubated at 37 °C for 16 h, at which point a zone of inhibition was clearly visible. 

### 4.7. Small-Volume Liquid Culture Antibiotic Activity Assay of 28ISP2-46

Strain 28ISP2-46^T^ was cultured in ISP2 broth (20 mL; 0.4% yeast extract, 1% malt extract, 0.4% dextrose, 3.3% ASW), for 15 days, at 28 °C, 180 rpm. The cells were harvested from 10 mL of culture using centrifugation. The supernatant (10 mL) was extracted with ethyl acetate (1:1, *v*/*v*). The remaining cell pellet was extracted with 1 mL methanol, vortexed for 1 min, and cell debris was removed by centrifugation at 13,000× *g*, 5 min. An aliquot of supernatant (0.5 mL) was treated with proteinase K (100 µg/mL, 1.5 h, 37 °C). All the samples were tested for antibiotic activity against the human pathogen *S. aureus* Newman, a strain that has been widely used as a model for staphylococcal infection. *S. aureus* Newman was cultured in Mueller–Hinton broth, 37 °C, for 16 h, then diluted in fresh Mueller–Hinton broth to OD_600_ = 0.1 and spread (25 µL) on a Mueller–Hinton agar plate. Wells were punched in the agar using the base of a sterile 1 mL pipette tip. Each of the extracts (50 µL) was added to a well. The activity of the extracts was compared to controls of a disk containing 5 µg of levofloxacin and a well containing 10 µg vancomycin. The plates were incubated at 37 °C for 16 h, at which point zones of inhibition could be observed.

### 4.8. Analysis and Identification of the Active Compound

Strain 28ISP2-46^T^ was cultured in 25% ISP2 broth (×2 1 L) in Erlenmayer 2 L flasks for 15 days, at 28 °C, 180 rpm. The culture broth was extracted using ethyl acetate (1:1, *v*/*9-*) three times and the combined extracts were dried over anhydrous MgSO_4_ and evaporated in vacuo to give a crude extract, which was subjected to liquid chromatography mass spectrometry (LC–MS) analysis (Supplementary Section S1).

### 4.9. Antimicrobial Susceptibility Testing of the Active Compound

Antimicrobial susceptibility testing (AST) employed *A. baumannii* ATCC 17978, *A. baumannii* CCD167h1.1, a derivative of ATCC 17,978 with deletion of two efflux pumps (Δ*adeIJ* ΔA1S_3447-3446), *E. coli* BW25113, *E. coli* CCD121a8, a derivative of BW25113 with deletion of six efflux pumps (Δ*acrAB* Δ*acrD* Δ*emrAB* Δ*macAB* Δ*mdfA* Δ*mdtK*), *E. coli* CCD181-1 (BW25113 imp4213) with increased membrane permeability, *K. pneumoniae* ATCC 10031, *P. aeruginosa* ATCC 33359, *P. aeruginosa* CCD047, a derivative of NCTC 11,451 with deletion of four efflux pumps (Δ*mexAB*, Δ*mexCD*, Δ*mexVW*, Δ*mexXY*) and *S. aureus* SH1000. AST was based on the guidelines described in the Clinical and Laboratory Standards Institute (CLSI) as follows. Compound **2** and the control antibiotic sitafloxacin were diluted in DMSO and stored at room temperature. Working stocks were prepared by making 10-point, 2-fold serial dilutions in DMSO at 50× the desired final concentration and 4 µL of diluted samples were added to wells of a 96-well microtiter plate (Greiner Bio-One). Final concentration ranges were 0.2 to 200 µM for the novel compounds and 0.02 to 10 µM for sitafloxacin with two columns of the 96-well plates reserved for the growth control and the negative control containing only cation-adjusted Mueller–Hinton broth (CAMHB).

Bacterial strains were streaked from frozen stocks onto Mueller–Hinton agar (MHA) to obtain individual colonies. Overnight cultures were prepared by suspending 3–4 colonies in CAMHB and incubating statically at 37 °C. Overnight cultures were diluted 1:100 in CAMHB except for *E. coli* BW25113 (1:1000) and BW25113 imp4213 (1:10) and incubated at 37 °C for 1 h. Cultures were then diluted 1:100 in prewarmed CAMHB and microtitre plates inoculated with 196 µL per well (final inoculum ~105 CFU/mL). After inoculation, plates were covered with transparent seals (Greiner Bio-One) or, for *A. baumannii*, breathable seals (Sigma Aldrich) and incubated at 37 °C for 20–24 h. OD_600_ was read (Perkin Elmer EnVision Microplate Reader) and the minimum inhibitory concentrations (MICs) determined as the minimum concentration of compound required to reduce OD_600_ by 90% as compared to no-compound controls.

### 4.10. Cytotoxicity Assay 

Hep G2 cells (ATCC HB-8065) were seeded at a density of 2 × 10^4^ cells/well in a 96-well, cell culture-treated microplate. They were incubated for 24 h at 37 °C in 5% CO_2_ to allow the cells to adhere. Cells were then exposed to a 2-fold serial dilution series of compound **2**, in duplicate, giving a final concentration range of 0.2–100 µM. Thioridazine hydrochloride (Sigma Aldrich) was used as a positive control. The viability of the cells was determined, after a further 24 h incubation, using CellTiter-Glo (Promega) according to the manufacturer’s instructions. Inhibition curves were generated to give the concentration of compound inhibiting 50% of cell viability (IC_50_) and percentage viability of the cells at the top tested concentration (100 µM).

### 4.11. Genome Sequencing

Genomic DNA from strain 28ISP2-46^T^ was purified with a GenElute™ Bacterial Genomic DNA Kit (Sigma-Aldrich) with some alterations to the manufacturer’s instructions as follows. 28ISP2-46^T^ was grown for 3 weeks to confluence, in 50 mL ISP2, at 28 °C. The cells were collected using centrifugation and the resulting pellet was frozen at −70 °C. The frozen pellet was crushed using a sterile pestle and mortar. The crushed mass was transferred to an Eppendorf tube, 200 µL of a lysozyme solution (45 mg/mL) was added, and the tube was inverted to mix and then incubated at 37 °C for 60 min. RNAase solution (Sigma-Aldrich, R6148) was added (as per the manufacturer’s instructions) and the tube incubated for 2 min at 20 °C. Then Proteinase K (200 µg/mL) and 200 µL lysis buffer (provided in the kit) were added, the tube inverted 3 times and then incubated at 52 °C for 20 min. Ethanol (100%, 200 µL) was added to the tube, which was inverted to mix. To remove the cell debris from the DNA, the tube was centrifuged (5 min, 5000× *g*). Using a wide bore pipette the supernatant was transferred to a spin column and the DNA purified (as per the GenElute™ Bacterial Genomic DNA Kit instructions). The DNA concentration and purity were ascertained by measuring the absorbance at 260 nm, and any DNA shearing was identified by gel electrophoresis. 

The Bristol Genomics Facility carried out quality assessment and normalisation of amplicon pools for the genomic DNA sample. Using 1 µg of DNA, a paired-end TruSeq library was generated, indexed with a Nextera^®^ XT Index Kit and shotgun sequenced on an Illumina MiSeq, producing 2 × 300 bp reads. The same genomic DNA sample (1 µg) was also sequenced using a R9 MinION flow cell indexed with the EXP-NBD103 kit (Oxford Nanopore Technologies, Oxford, UK), following the manufacturer’s instructions.

### 4.12. Genome Assembly and Mining

Nanopore sequence data were converted from FAST5 data to fastq files using Albacore 1.2.5 for base calling (Oxford Nanopore Technologies, Oxford, UK). Adaptor trimming and demultiplexing of the Nanopore reads were completed using Porechop (Porechop, RRID: SCR_016967). Adaptor and quality trimming of the Illumina reads was performed using Trim Galore v0.4.4 [41] with a PHRED score of 20 as a cut off and default parameters. Processed reads were either assembled with Unicycler v0.4.6 [16] or a combination of the long-read assembler Canu 1.8 [42] and the Unicycler pipeline. To maintain lower-length reads under 1000 bp Canu was run with the flag ‘stopOnReadQuality = false’. The Illumina only assembly was completed using the IDBA assembler v1.1.3 [43]. Alignment of trimmed reads against the final assemblies was conducted using Bowtie2 v2.2.9 [44]. The draft genome was mined using antiSMASH 5.0 (“Antibiotic and Secondary Metabolites Analysis Shell”) [21]. The phylogenetic tree was produced using the Type Strain Genome Server (TYGS) [19] and drawn using Interactive Tree of Life (iTOL) [45].

### 4.13. Genome BGC Comparison

Genomes were annotated using antiSMASH 5.0 [21] with relaxed detection strictness and all extra features on. The BGCs with similarity to the kosinostatin BGC were manually annotated using BLASTp to search for homologous proteins for each translated ORF, and this annotation was extended 20 ORFs upstream and downstream of the antiSMASH-defined BGC boundaries. ORFs were then aligned with kosinostatin BGC genes with homologous functions using BLASTp, and annotated as *kstA-D*, *kstRs*, *kstRg*, or *kstU* homologs. The nucleotide accession number for the *Micromonospora* sp. TP-A0468 kosinostatin BGC is JN038178.1. For the kosinostatin ClusterBLAST [21] relatives, GenBank assembly accession numbers are as follows: *Actinokineospora bangkokensis* 44EHW (GCA_001940455.1), *Micromonospora haikouensis* DSM 45,626 (GCA_900091595.1), *Nocardiopsis alkaliphila* YIM 80,379 (GCA_000341005.1), *Nocardiopsis valliformis* DSM 45,023 (GCA_000340985.1), *Streptomyces* sp. ScaeMP-6W (GCA_900091885.1), *Streptomyces* sp. SM8 (GCA_000299175.2). 

The genomes of the type Ia *Micromonospora* species were annotated using antiSMASH 5.0 [21] and manually inspected for BGCs with significant similarity to each BGC of strain 28ISP2-46^T^.

For the group Ia *Micromonospora* species, GenBank assembly accession numbers are as follows: *Micromonospora aurantiaca* ATCC 27,029 (GCA_000145235.1), *Micromonospora auratinigra* DSM 44,815 (GCA_900089595.1), *Micromonospora chaiyaphumensis* DSM 45,246 (GCA_900091435.1), *Micromonospora chalcea* DSM 43,026 (GCA_002926165.1), *Micromonospora chersina* DSM 44,151 (GCA_900091475.1), *Micromonospora humi* DSM 45,647 (GCA_900090105.1), *Micromonospora marina* DSM 45,555 (GCA_900091565.1), *Micromonospora sediminicola* DSM 45,794 (GCA_900089585.1) and *Micromonospora tulbaghiae* CNY-010 (GCA_003612775.1).

## Figures and Tables

**Table 1:** MIC_90_ of isoquinocycline B (**2**), purified from strain 28ISP2-46^T^. Sitafloxacin was utilised as a positive control. The experiment was performed twice (error values in parentheses are standard error from the mean and are only shown for experiments where the MIC_90_ values from the two experiments are not identical).

Strain	MIC_90_ (µM)	
	2	Sitafloxacin
*A. baumannii* ATCC 17978	25	0.0781
*A. baumannii* efflux KO ^a^	1.563	<0.0195
*P. aeruginosa* ATCC 33359	>100.0	0.469 (±0.156)
*P. aeruginosa* efflux KO ^b^	12.5	0.156
*E. coli* BW25113	>100.0	<0.0195
*E. coli* efflux KO ^c^	9.375 (±3.125)	<0.0195
*E. coli* IMP ^d^	<0.1953	<0.0195
*K. pneumoniae* ATCC 10031	12.5	<0.0195
*S. aureus* SH1000	1.562	0.117 (±0.039)

^a^ *A. baumannii* CCD167h1.1, efflux KO (ATCC 17978 Δ*adeIJ* Δ*A1S_3447-3446*); ^b^ *P. aeruginosa* CCD047, efflux KO (NCTC 11,451 Δ*mexAB* Δ*mexCD* Δ*mexVW* Δ*mexXY*); ^c^ *E. coli* CCD121a8, efflux KO (BW25113 Δ*acrAB* Δ*acrD* Δ*emrAB* Δ*macAB* Δ*mdfA* Δ*mdtK*); ^d^ *E. coli* CCD181-1, increased membrane permeability (BW25113 *imp4213*).

**Table 2:** Cytotoxicity assay of isoquinocycline B (**2**) against the HepG2 (ATCC HB-8065) cell line. Thioridazine hydrochloride was utilised as a positive control.

Compound ID	IC_50_ (µM)	Cell Viability at 100 µM (%)
**2**	13.3	0.0
CONTROL	22.5	0.0

**Table 3:** Summary statistics for assemblies from Illumina only, Oxford Nanopore only and Illumina/Oxford Nanopore hybrid genome sequencing of strain 28ISP2-46^T^.

	Illumina Only	Nanopore Only	Illumina/Nanopore Assembly
Number of contigs	637	196	1
Total length (Mb)	6.62	8.06	6.64
Shortest contig	123 nt	1.03 kb	6.64 Mb
Largest contig	1.36 kb	6.70 Mb	6.64 Mb
Average coverage ^a^	36.3	313	290
Coverage std dev	19.9	137	113
N50 (kb)	22.3	6700	6640
L50	92	1	1
Mapped reads	98.5%	99.6%	98.5%
Error rate	1.27%	10.8%	0.44%
GC content	72.4%	72.3%	72.5%

^a^ Coverage is average reads per base, calculated from an alignment of the trimmed reads (reads trimmed with TrimGalore, aligned against the assembly contigs using Bowtie2). Average coverage is the unweighted average of the coverage of each contig separately.

**Table 4:** Biosynthetic gene clusters identified in the genome of strain 28ISP2-46^T^ using AntiSMASH.

Region	Type	Cluster Size (kb)	Most Similar Known Cluster	Strain of Most Similar Known Cluster	Similarity ^a^
1	T3PKS	40.6	Alkyl-O-dihydrogeranyl-methoxyhydroquinones	*Actinoplanes missouriensis* 431	71%
2	Terpene	19.8	Isorenieratene	*Streptomyces argillaceus*	25%
3	Terpene	16.8	Phosphonoglycans	*Glycomyces* sp. NRRL B-16210	3%
4	NAGGN	14.7	*No similar cluster*		
5	NRPS, T1PKS	61.7	Bleomycin	*Streptomyces verticillus*	6%
6	Lanthipeptide	21.5	*No similar cluster*		
7	NRPS, T1PKS	66.3	Nostopeptolide	*Nostoc* sp. GSV224	25%
8	Lanthipeptide	21.6	SapB	*Streptomyces coelicolor* A3(2)	75%
9	T1PKS, NRPS-like	214	Rifamycin	*Salinispora arenicola* CNS-205	38%
10	NRPS, arylpolyene	84.8	Kedarcidin	*Streptoalloteichus* sp. ATCC 53650	13%
11	Terpene	19.3	Nocathiacin	*Nocardia sp. ATCC 202099*	4%
12	T2PKS	71.2	Formicamycins A-M	*Streptomyces* sp. KY5	13%
13	NRPS	49.1	Azicemicin	*Kibdelosporangium* sp. MJ126-NF4	13%
14	Siderophore	11.8	Desferrioxamine	*Streptomyces* sp. ID38640	100%
15	Oligosaccharide, terpene, lanthipeptide	51.0	Lobosamide	*Micromonospora* sp. RL09-050-HVF-A	13%
16	NRPS, T3PKS	114	Feglymycin	*Streptomyces* sp. DSM 11171	63%
17	T2PKS, ectoine, NRPS, T1PKS, other	158	Kosinostatin	*Micromonospora* sp. TP-A0468	77%
18	Bacteriocin	10.8	Lymphostin	*Salinispora tropica* CNB-440	33%
19	Terpene	21.0	*No similar cluster*		

^a^ The percentage of genes in the cluster with homologues in the ‘most similar known cluster’.

**Table 5:** The predicted functions for the groups of kosinostatin biosynthetic genes as defined by Ma et al. [22].

Gene Group	Predicted Function	Colour in Figure 6
kstA	Synthesise the anthracycline and modify its rings.	Red
kstB	Load nicotinic acid and convert it to a pyrrole.	Yellow
kstC	Convert the pyrrole and phosphoribosyl pyrophosphate to the pyrrolopyrrole.	Green
kstD	Synthesise the branched deoxy-octose and attach it to the anthracycline.	Blue
kstRg	Transcription factors that control the expression of the BGC.	Black
kstRs	Provide self-resistance to kosinostatin.	White
kstU	Not involved in kosinostatin biosynthesis.	Grey

## Data Availability

Further data are available on request from the corresponding authors.

## Supplementary Materials

The following are available online at https://www.mdpi.com/1660-3397/19/2/105/s1. Supplementary Section S1: LC–MS analysis of the extract from 28ISP2-28^T^; Supplementary Section S2: NMR chemical shifts of (2) (isoquinocycline B); Supplementary Section S3: 16S rRNA gene sequencing of 28ISP2-46T; Figure S1: Photographs of sponge sample B0078 retrieved from the Knipovich seamount; Figure S2: Small-volume liquid culture antibiotic activity assay of 28ISP2-28T; Figure S3: 1H NMR spectrum of isoquinocycline B (2, CD_3_OD, 500 MHz); Figure S4: 13C NMR spectrum of isoquinocycline B (2, CD_3_OD, 125 MHz); Figure S5: 1H-1H COSY spectrum of spectrum of isoquinocycline B (2); Figure S6: HSQC spectrum of isoquinocycline B (2); Figure S7: HMBC spectrum of isoquinocycline B (2); Figure S8: The predicted function of KstB7 in pyrrolopyrrole biosynthesis according to Ma et al. 2013 [22]; Figure S9: The WebMGA COG families and the fractional coverage of each COG family for ORFs in the *Micromonospora* sp. 28ISP2-46^T^ genome; Table S1: Bacterial strains used in this study; Table S2: Sequence identity comparison of the 28ISP2-46^T^ 16S rRNA gene sequence with the most closely related strains; Table S3: BCG comparison of Micromonospora group Ia strains with strain 28ISP2-46T; Table S4: The NCBI Prokaryotic Genome Annotation Pipeline Annotation data for the strain 28ISP2-46^T^ genome assembly.
