# Correction: Back et al. A New *Micromonospora* Strain with Antibiotic Activity Isolated from the Microbiome of a Mid-Atlantic Deep-Sea Sponge. *Mar. Drugs* 2021, *19*, 105

**DOI:** 10.3390/md21040214

**Published:** 2023-03-28

**Authors:** Catherine R. Back, Henry L. Stennett, Sam E. Williams, Luoyi Wang, Jorge Ojeda Gomez, Omar M. Abdulle, Thomas Duffy, Christopher Neal, Judith Mantell, Mark A. Jepson, Katharine R. Hendry, David Powell, James E. M. Stach, Angela E. Essex-Lopresti, Christine L. Willis, Paul Curnow, Paul R. Race

**Affiliations:** 1 School of Biochemistry, University of Bristol, University Walk, Bristol BS8 1TD, UK; 2 School of Chemistry, University of Bristol, Cantock’s Close, Bristol BS8 1TS, UKchris.willis@bristol.ac.uk (C.L.W.); 3 Summit Therapeutics, Merrifield Centre, Rosemary Lane, Cambridge CB1 3LQ, UK; 4 Woolfson Bioimaging Facility, University of Bristol, University Walk, Bristol BS8 1TD, UKm.a.jepson@bristol.ac.uk (M.A.J.); 5 School of Earth Sciences, University of Bristol, Wills Memorial Building, Queens Road, Bristol BS8 1RJ, UK; 6 School of Natural and Environmental Sciences, Newcastle University, King’s Road, Newcastle upon Tyne NE1 7RU, UK; 7 Defence Science and Technology Laboratory, Porton Down, Salisbury, Wiltshire SP4 0JQ, UK

## Text Correction

After publication of this article [1], it came to our attention that the proposed species name *Micromonospora ferruginea* sp. nov. contravenes Rule 12b of the International Code of Nomenclature of Prokaryotes as the name *Micromonospora echinospora* subsp. *ferruginea* Luedemann and Brodsky 1964 (Approved Lists 1980) was validly published. In this corrigendum, we thus propose replacing *Micromonospora ferruginea* with the name *Micromonospora robiginosa* sp. nov. The description of this species is as follows:

*Micromonospora robiginosa* sp. nov. (ro.bi.gi.no’sa. L. fem. adj. *robiginosa*, referring to the colour of iron rust, dark red, from the colour of the bacterial colonies growing on ISP2 agar). *M. robiginosa* is an aerobic, Gram-positive, non-motile, mesophilic actinomycete that forms hard, rust-red-coloured colonies on ISP2 agar. It produces a peach-pink diffusible pigment, particularly visible on pale-coloured media. Substrate hyphae are extensively branched and fragmented irregularly into rod-shaped, non-motile elements. It produces spores that are ovoid to spherical, slightly rough, non-motile, and approximately 1 µm in diameter once fully grown. Growth of the strain is between 20 and 37 °C, between pH 5 and 9, and in the presence of up to 4% NaCl on unbuffered Luria–Bertani agar. The strain grows optimally at 28 °C, at pH 7, and in the presence of 0% NaCl. The genome size of the strain type is 6.64 Mb, and its DNA G+C content is 72.5 mol %. The strain type 28ISP2-46^T^ (NCIMB 15402^T^, DSM 111791^T^) was isolated from a deep-sea sponge from the Knipovich Seamount in the Atlantic Ocean. 

The authors state that the scientific conclusions are unaffected. This correction was approved by the Academic Editor. The original publication has also been updated.
