# Supplementary material for: A New Micromonospora Strain with Antibiotic Activity Isolated from the Microbiome of a Mid-Atlantic Deep-Sea Sponge
Source: Mar Drugs. 2021 Feb 11;19(2):105. doi: 10.3390/md19020105 (PMC7918784; doi:10.3390/md19020105)
Supplement: Supplementary file 1 [file marinedrugs-19-00105-s001.pdf]

Supplementary Materials

A new *Micromonospora* strain with potent antibiotic activity isolated from the microbiome of a mid-Atlantic deep-sea sponge

Catherine R. Back, Henry L. Stennett, Sam E. Williams, Luoyi Wang, Jorge Ojeda Gomez, Omar M. Abdulle, Thomas Duffy, Christopher Neal, Judith Mantell, Mark A. Jepson, Katharine R. Hendry, David Powell, James E. M. Stach, Angela E. Essex-Lopresti, Christine L. Willis, Paul Curnow and Paul R. Race.

Contents:

Supplementary Sections S1-3

Supplementary Figures S1-7

Supplementary Tables S1-3

Supplementary References

Supplementary Section S1: LCMS analysis of the extract from 28ISP2-28[T]

LCMS data were obtained on a Waters LCMS system comprising Waters 2767 autosampler, Waters 515 HPLC pump, Waters 2998 Diode Array detector, Waters 2424 ELS detector and Waters Quatro Micro mass spectrometer. HPLC grade H_2O and MeCN were added with 0.05% formic acid as solvent system. Analytical LC-MS data were obtained using a Phenomenex Kinetex column (C18, 250 x 4.60 mm, 5 μm) at a flow rate of 1 mL/min, with a gradient of 5% MeCN to 95% MeCN in 20 mins. Preparative HPLC purification were carried out using a Phenomenex Kinetex column (C18, 250 x 21.20 mm, 5 μm) at a flow rate of 16 mL/min, with a gradient of 30 to 40% of MeCN in water over 20 min. HR-ESI-MS data were obtained on a Bruker Daltonics micrOTOF II instrument. NMR data were collected on a Bruker Cryo500 NMR spectrometer.

Supplementary Section S2: NMR chemical shifts of (**2**) (Isoquinocycline B).

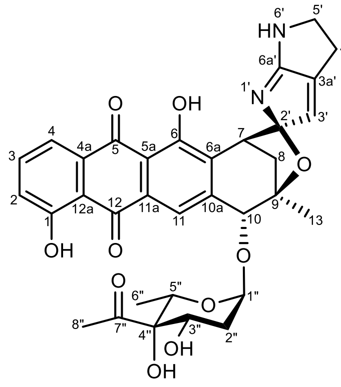

1

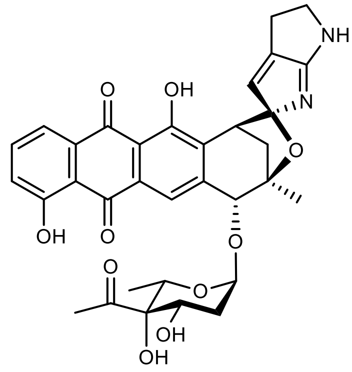

2

HR-ESI-MS: m/z = 617.2143 $[M+H]^+$ (617.2130 calcd for $C_{33}H_{33}N_2O_{10}$); ^1H NMR (500 MHz, methanol-d_4): δ 7.94 (s, 1H, H-11), 7.87 (dd, J = 7.6, 1.1 Hz, 1H, H-4), 7.79 (t, J = 8.0 Hz, 1H, H-3), 7.37 (dd, J = 8.4, 1.1 Hz, 1H, H-2), 5.57 – 5.51 (m, 1H, H-3′), 5.44 (dd, J = 5.2, 3.4 Hz, 1H, H-1″), 4.70 – 4.63 (m, 1H, H-5′), 4.58 (d, J = 3.2 Hz, 1H, H-10), 4.23 (d, J = 3.8 Hz, 1H, H-7), 4.16 (m, 2H, h-5′), 3.85 (dd, J = 6.0, 3.8 Hz, 1H, H-3″), 2.89 – 2.78 (m, 2H, H-4′), 2.51 – 2.36 (m, 2H, H-8), 2.26 (s, 3H, H-8″), 2.23 (m, 1H, H-2″), 1.86 (ddd, J = 14.5, 6.0, 3.4 Hz, 1H, H-2″), 1.73 (s, 3H, H-13), 1.15 (d, J = 6.5 Hz, 3H, H-6″); ^{13}C NMR (125 MHz, methanol-d_4): δ 212.9 (C-7″), 189.3 (C-5), 188.7 (C-12), 169.2 (C-6a′), 163.9 (C-1), 159.8 (C-6), 145.5 (C-10a), 141.8 (C-3a′), 138.2 (C-3), 134.7 (C-3′), 134.6 (C-4a), 134.2 (C-6a), 133.8 (C-11a), 126.0 (C-2), 121.4 (C-11), 120.4 (C-4), 117.2 (C-12a), 116.4 (C-5a), 116.0 (C-2′), 97.6 (C-1″), 87.0 (C-9), 82.0 (C-4″), 78.3 (C-10), 71.3 (C-3″), 67.0 (C-5″), 54.7 (C-5′), 45.0 (C-7), 35.9 (C-8), 33.7 (C-2″), 27.0 (C-8″), 23.4 (C-13), 21.8 (C-4′), 14.9 (C-6″).

Supplementary Section S3: 16S rRNA gene sequencing of 28ISP2-46^{T}.

Forward 16S rRNA gene sequencing

```
TNNNNNNGCAGTCGAGCGGAAGGNCCTTCGGGNNANTCGAGCGGCGAACGGGTGAGTAAC
ACGTGAGCAACCTGCCCTAGGCTTTGGGATAACCCCGGGAAACCGGGGCTAATACCGAAT
ATGACTACTGATCGCATGGTTGGTGGTGGAAAGTTTTTCGGCCTGGGATGGGCTCGCGGC
CTATCAGCTTGTTGGTGGGGTGATGGCCTACCAAGGCGACGACGGGTAGCCGGCCTGAGA
GGGCGACCGGCCACACTGGGACTGAGACACGGCCCAGACTCCTACGGGAGGCAGCAGTGG
GGAATATTGCACAATGGGCGGAAGCCTGATGCAGCGACGCCGCGTGAGGGATGACGGCCT
TCGGGTTGTAAACCTCTTTCAGCAGGGACGAAGCGTAAGTGACGGTACCTGCAGAAGAAG
CGCCGGCCAACTACGTGCCAGCAGCCGCGGTAAGACGTAGGGCGCGAGCGTTGTCCGGAT
TTATTGGGCGTAAAGAGCTCGTAGGCGGCTTGTCGCGTCGACCGTGAAAACCTGGGGCTC
AACTCCAGGCCTGCGGTCGATACGGGCAGGCTAGAGTTCGGTAGGGGAGACTGGAATTCC
TGGTGTAGCGGTGAAATGCGCAGATATCAGGAGGAACACCGGTGGCGAAGGCGGGTCTCT
GGGCCGATACTGACGCTGAGGAGCGAAAGCGTGGGGAGCGAACAGGATTAGATACCCTGG
TAGTCCACGCTGTAAACGTTGGGCGCTAGGTGTGGGGGGCCTCTCCGGTTCCCTGTGCCG
CAGCTAACGCATTAAGCGCCCCGCCTGGGGAGTACGGCCGCAAGGCTAAAACTCAAAGGA
ATTGACGGGGGCCCGCACAAGCGGCGGAGCATGCGGATTAATTCGATGCAACGCGANN
```

Reverse 16S rRNA gene sequencing

```
ACGGTACCTGCAGAAGAAGCGCCGGCCAACTACGTGCCAGCAGCCGCGGTAAGACGTAGGGCGCGAGCGT
TGTCCGGATTTATTGGGCGTAAAGAGCTCGTAGGCGGCTTGTCGCGTCGACCGTGAAAACCTGGGGCTCA
ACTCCAGGCCTGCGGTCGATACGGGCAGGCTAGAGTTCGGTAGGGGAGACTGGAATTCCTGGTGTAGCGG
TGAAATGCGCAGATATCAGGAGGAACACCGGTGGCGAAGGCGGGTCTCTGGGCCGATACTGACGCTGAGG
AGCGAAAGCGTGGGGAGCGAACAGGATTAGATACCCTGGTAGTCCACGCTGTAAACGTTGGGCGCTAGGT
GTGGGGGGCCTCTCCGGTTCCCTGTGCCGCAGCTAACGCATTAAGCGCCCCGCCTGGGGAGTACGGCCGC
AAGGCTAAAACTCAAAGGAATTGACGGGGGCCCGCACAAGCGGCGGAGCATGCGGATTAATTCGATGCAA
CGCGAAGAACCTTACCTGGGTTTGACATGGCCGCAAAACTTGCAGAGATGTAAGGTCCTTCGGGGGCGGT
CACAGGTGGTGCATGGCTGTCGTCAGCTCGTGTCGTGAGATGTTGGGTTAAGTCCCGCAACGAGCGCAAC
CCTCGTTCGATGTTGCCAGCGCGTTATGGCGGGGACTCATCGAAGACTGCCGGGGTCAACTCGGAGGAAG
GTGGGGATGACGTCAAGTCATCATGCCCCTTATGTCCAGGGCTTCACGCATGCTACAATGGCCGGTACAA
TGGGCTGCGATACCGTGAGGTGGAGCGAATCCCAAAAAGCCGGTCTCAGTTCGGATCGGGGTCTGCAACT
CGACCCCGTGAAGTCGGAGTCGCTAGTAATCGCAGATCAGCAACGCTGCGGTGAATACGTTCCCGGGCCT
TGTACACACCGCCCGTCACGTCACGAAAGTCGGCAACACCCGAAGCCGGTNGCCCAACCCTTGNGGAGGG
AGCCGTNGAAG
```

16S rRNA gene sequencing (combined forward and reverse)

```
GCAGTCGAGCGGAAGGNCCTTCGGGNNANTCGAGCGGCGAACGGGTGAGTAACACGTGAGCAACCTGCCC
TAGGCTTTGGGATAACCCCGGGAAACCGGGGCTAATACCGAATATGACTACTGATCGCATGGTTGGTGGT
GGAAAGTTTTTCGGCCTGGGATGGGCTCGCGGCCTATCAGCTTGTTGGTGGGGTGATGGCCTACCAAGGC
GACGACGGGTAGCCGGCCTGAGAGGGCGACCGGCCACACTGGGACTGAGACACGGCCCAGACTCCTACGG
GAGGCAGCAGTGGGGAATATTGCACAATGGGCGGAAGCCTGATGCAGCGACGCCGCGTGAGGGATGACGG
CCTTCGGGTTGTAAACCTCTTTCAGCAGGGACGAAGCGTAAGTGACGGTACCTGCAGAAGAAGCGCCGGC
CAACTACGTGCCAGCAGCCGCGGTAAGACGTAGGGCGCGAGCGTTGTCCGGATTTATTGGGCGTAAAGAG
CTCGTAGGCGGCTTGTCGCGTCGACCGTGAAAACCTGGGGCTCAACTCCAGGCCTGCGGTCGATACGGGC
AGGCTAGAGTTCGGTAGGGGAGACTGGAATTCCTGGTGTAGCGGTGAAATGCGCAGATATCAGGAGGAAC
ACCGGTGGCGAAGGCGGGTCTCTGGGCCGATACTGACGCTGAGGAGCGAAAGCGTGGGGAGCGAACAGGA
TTAGATACCCTGGTAGTCCACGCTGTAAACGTTGGGCGCTAGGTGTGGGGGGCCTCTCCGGTTCCCTGTG
CCGCAGCTAACGCATTAAGCGCCCCGCCTGGGGAGTACGGCCGCAAGGCTAAAACTCAAAGGAATTGACG
GGGGCCCGCACAAGCGGCGGAGCATGCGGATTAATTCGATGCAACGCGAAGAACCTTACCTGGGTTTGAC
ATGGCCGCAAAACTTGCAGAGATGTAAGGTCCTTCGGGGGCGGTCACAGGTGGTGCATGGCTGTCGTCAG
CTCGTGTCGTGAGATGTTGGGTTAAGTCCCGCAACGAGCGCAACCCTCGTTCGATGTTGCCAGCGCGTTA
TGGCGGGGACTCATCGAAGACTGCCGGGGTCAACTCGGAGGAAGGTGGGGATGACGTCAAGTCATCATGC
CCCTTATGTCCAGGGCTTCACGCATGCTACAATGGCCGGTACAATGGGCTGCGATACCGTGAGGTGGAGC
GAATCCCAAAAAGCCGGTCTCAGTTCGGATCGGGGTCTGCAACTCGACCCCGTGAAGTCGGAGTCGCTAG
TAATCGCAGATCAGCAACGCTGCGGTGAATACGTTCCCGGGCCTTGTACACACCGCCCGTCACGTCACGA
AAGTCGGCAACACCCGAAGCCGGTNGCCCAACCCTTGNGGAGGGAGCCGT
```

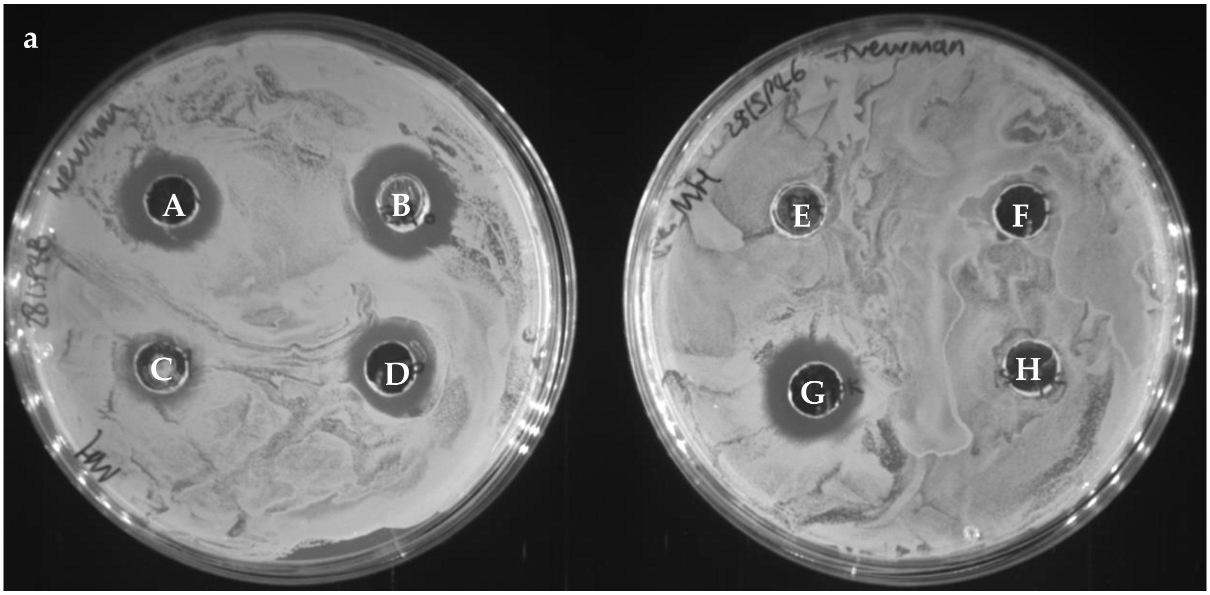

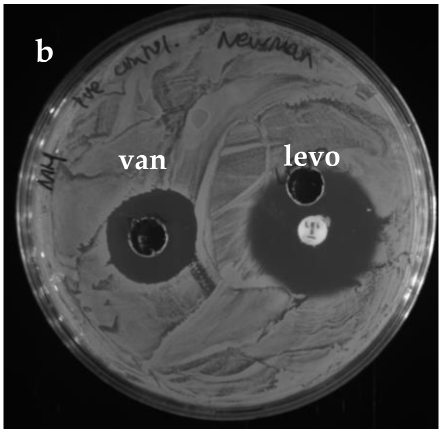

Figure S1. Small volume liquid culture antibiotic activity assay of 28ISP2-46^{T}. **(a)** Activity assay of extracts from 28ISP2-46^{T}: A, culture supernatant before extraction (50 µl); B, ethyl acetate fraction (50 µl); C, aqueous fraction (50 µl); D, pellet extraction with methanol (50 µl); E, ethyl acetate only control (50 µl); F, methanol only control (50 µl); G, culture supernatant before extraction treated with Proteinase K (50 µl); H, ISP2 media only treated with Proteinase K (50 µl). **(b)** Control plate with a well on the left containing 10 µg vancomycin (van) and a disk on the right containing 5 µg levofloxacin (levo).

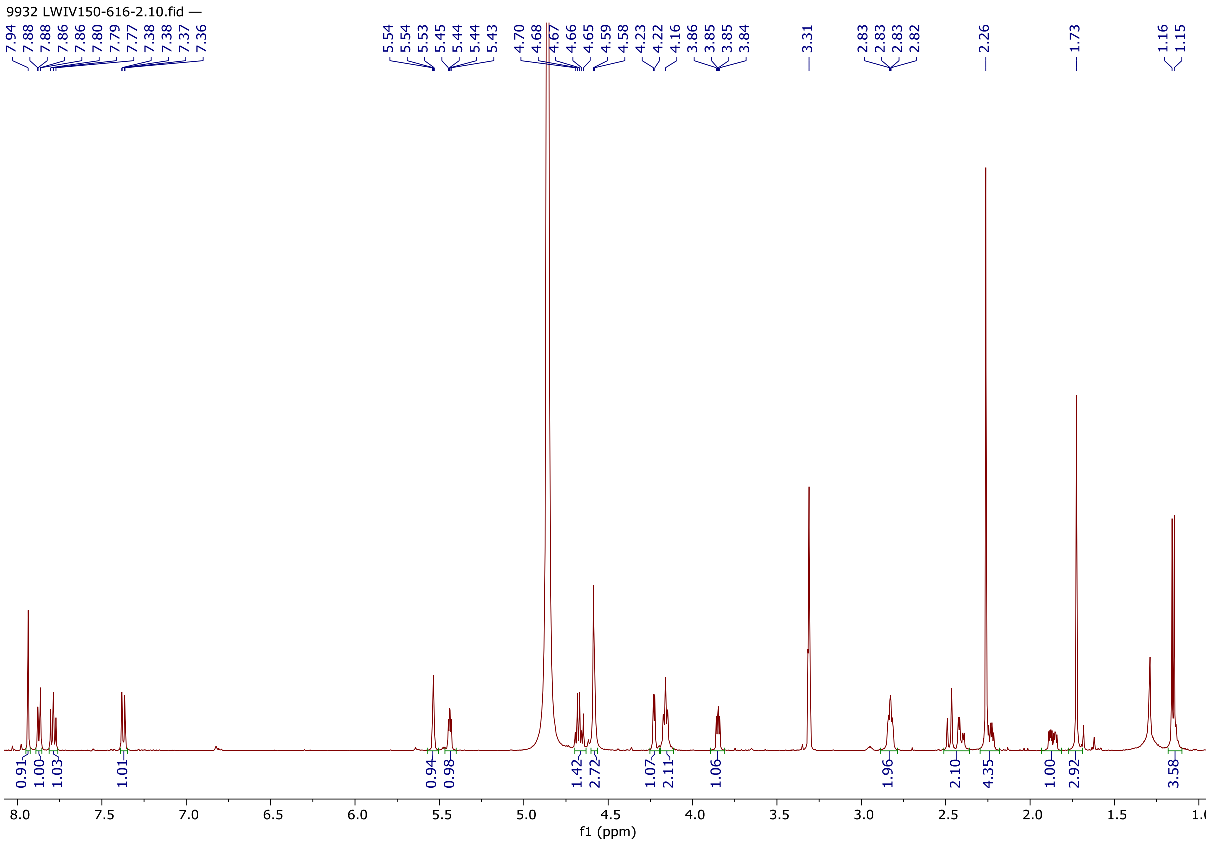

Figure S2. ^{1}H NMR spectrum of isoquinocycline B (**2**, CD_3OD, 500 MHz)

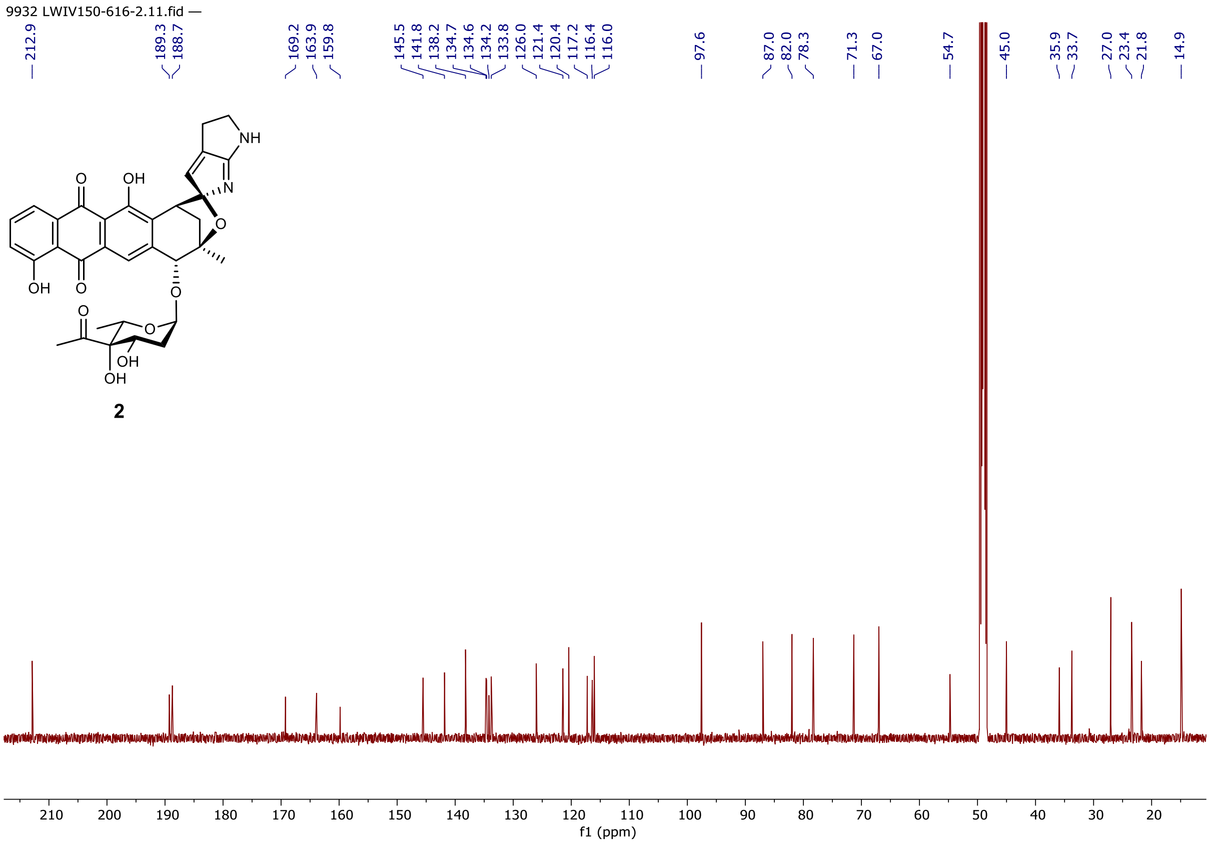

Figure S3. ^{13}C NMR spectrum of isoquinocycline B (**2**, CD_3OD, 125 MHz)

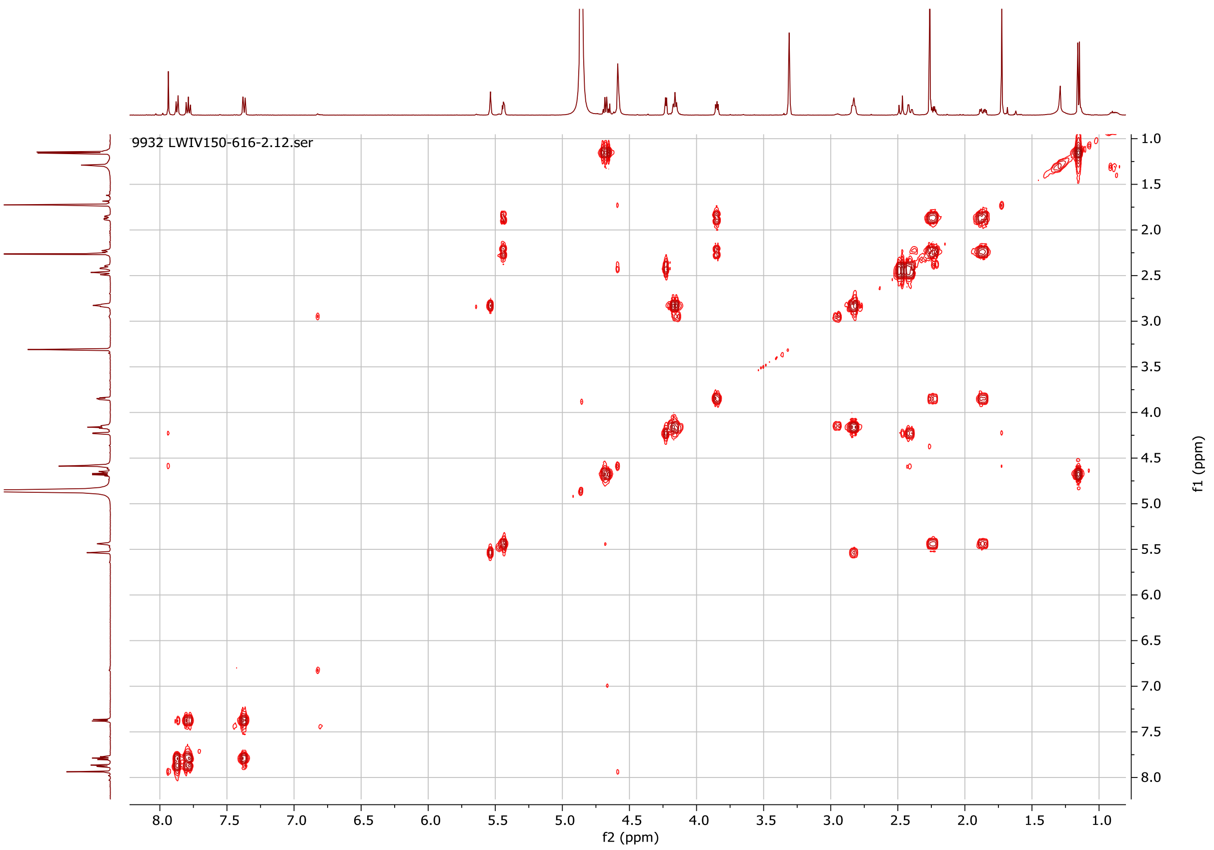

Figure S4. ^{1}H-^{1}H COSY spectrum of spectrum of isoquinocycline B (**2**)

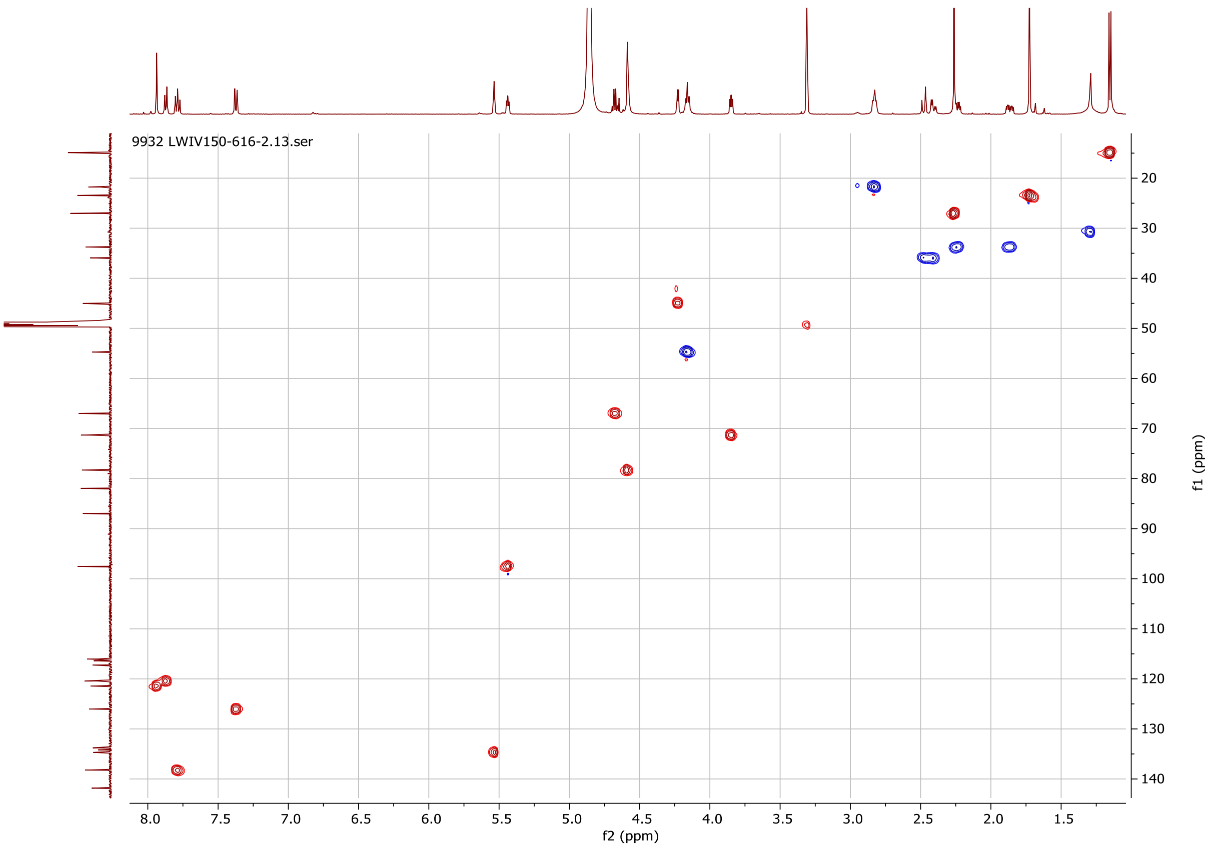

Figure S5. HSQC spectrum of isoquinocycline B (**2**)

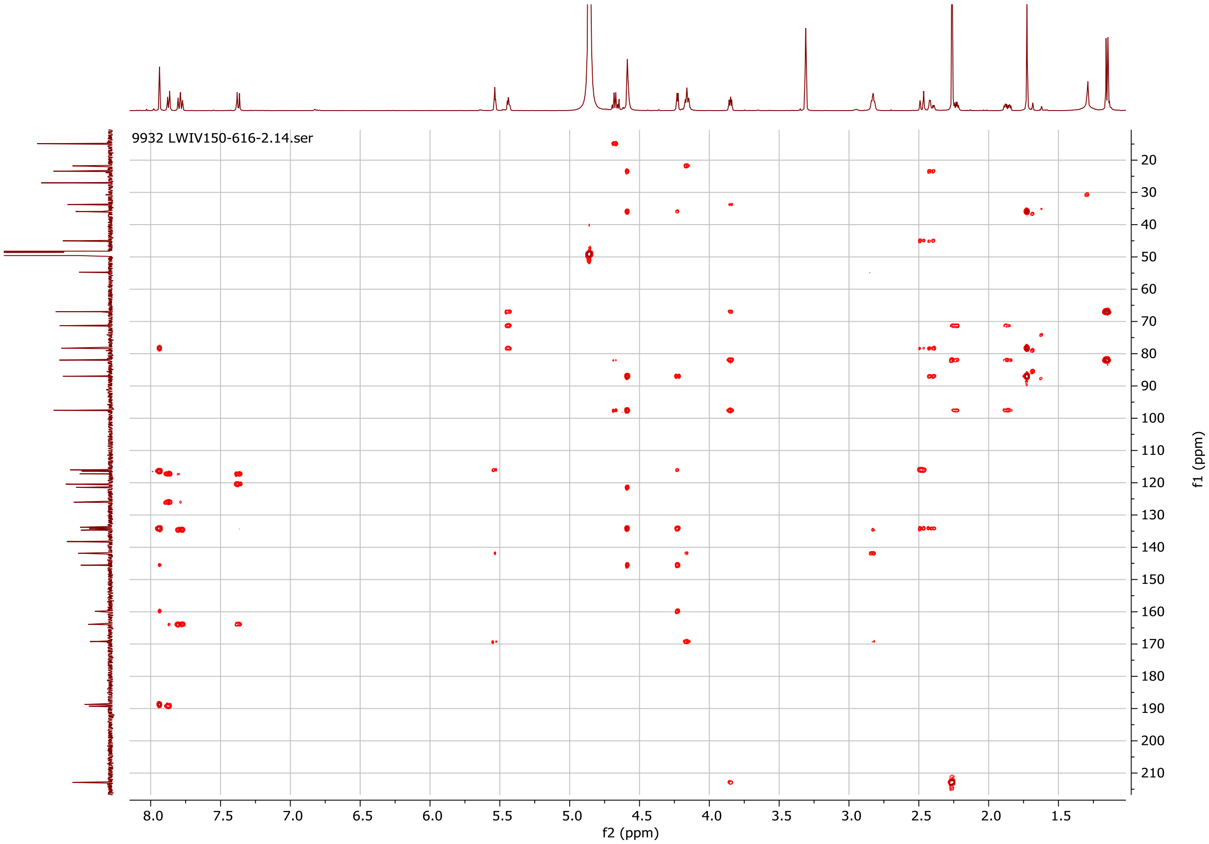

Figure S6. HMBC spectrum of isoquinocycline B (**2**)

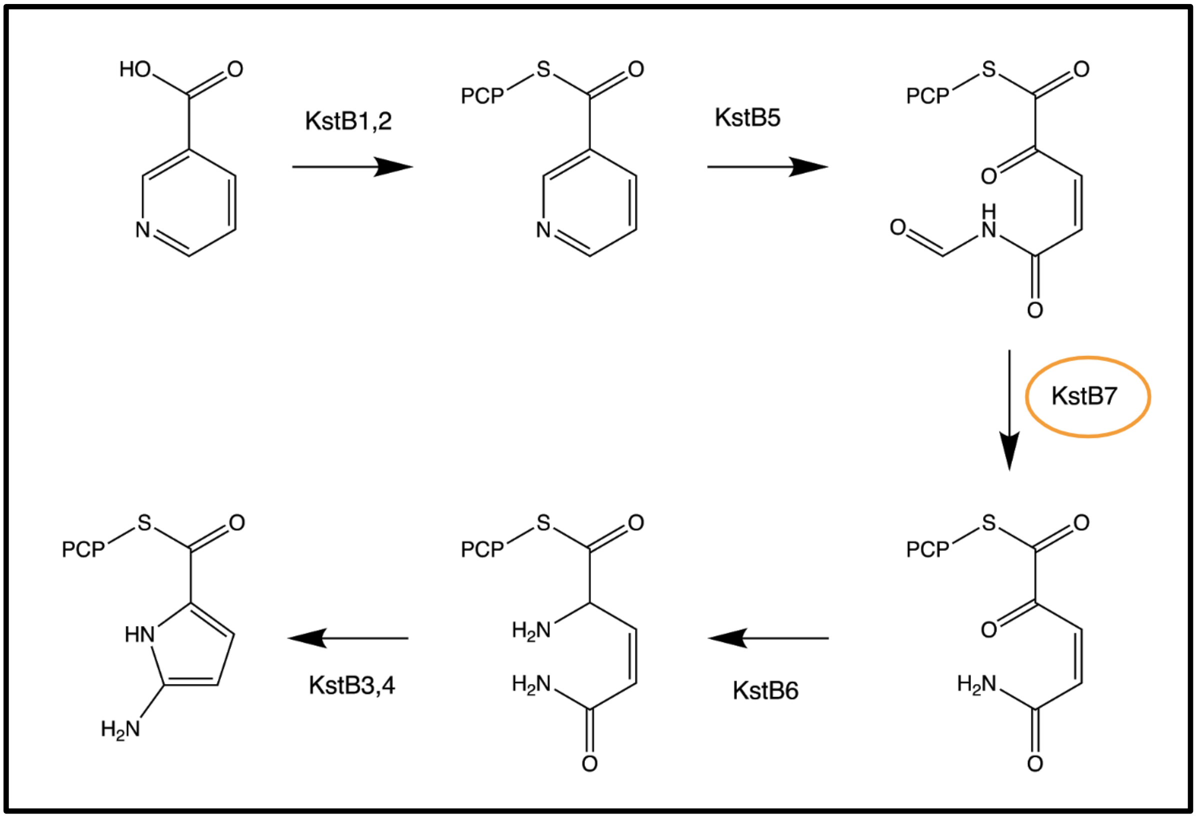

Figure S7. The predicted function of KstB7 in pyrrolopyrrole biosynthesis according to Ma *et al*. 2013 [1].

Table S1: Bacterial strains used in this study.

**Organism**	**Strain**	**Details**	**Reference**
28ISP2-46^T	*Micromonospora ferruginea* sp. nov.		This study
Staphylococcus aureus	Newman		[2]
S. aureus	Mu50	Methicillin & vancomycin resistant	[3]
Pseudomonas aeruginosa	PAO1		[4]
Escherichia coli	BW25113		[5]
Klebsiella pneumoniae	NCTC 5055		[6]
Acinetobacter baumannii	ATCC 19606		[7]
A. baumannii	ATCC 17978		[8]
A. baumannii	CCD167h1.1	ATCC 17978 Δ*adeIJ*, Δ*A1S_3447-3446*	
E. coli	CCD121a8	BW25113 Δ*acrAB*, Δ*acrD*, Δ*emrAB*, Δ*macAB*, Δ*mdfA*, Δ*mdtK*	
E. coli	CCD181-1	BW25113 *imp4213*	
K. pneumoniae	ATCC 10031		?
P. aeruginosa	ATCC 33359		[9]
P. aeruginosa	CCD047	NCTC 11451 Δ*mexAB*, Δ*mexCD*, Δ*mexVW*, Δ*mexXY*	
S. aureus	SH1000		[10]

Table S2: Sequence identity comparison of the 28ISP2-46^T 16S rRNA gene sequence with the most closely related strains. All *Micromonospora* strains.

Strain	Coverage (%)	Identity (%)
M. humi	99	99
M. maritima	99	99
M. marina	99	99
M. coxensis	99	99
M. aurantiaca	99	99
M. sediminicola	99	99
M. purpureochromogenes	99	99
M. chaiyaphumensis	99	99

Table S3. BCG comparison of *Micromonospora* group Ia strains (*M. humi*, *M. aurantiaca, M. auratinigra, M. chalcea, M. chaiyaphumensis, M. chersina, M. marina, M. sediminicola*, and *M. tulbaghiae)* with strain 28ISP2-46[T].

28ISP2-46[T] BGC region	BGC class	Closest BGC relative (genes with homologs)[a]	Group Ia species with homologous BGCs
1	Type III PKS	Alkyl-O-dihydrogeranyl-methoxy hydroquinones (71%)	All
2	Terpene	Isorenieratene (25%)	All
3	Terpene	Phosphonoglycans (3%)	All
4	N-acetylglutaminylglutamine amide (NAGGN)	None	All
5	NRPS / type I PKS	Bleomycin (6%)	All but *M. aurantiaca, M. marina, M. tulbaghiae*
6	Lanthipeptide	None	*M. chalcea*
7	NRPS, type I PKS	Nostopeptolide A2 (25%)	All but *M. sediminicola*
8	Lanthipeptide	SapB (75%)	*M. chaiyaphumensis, M. chersina, M. humi*
9	Type I PKS, NRPS-like	Rifamycin (38%)	*M. aurantiaca, M. chalcea, M. sediminicola*
10	NRPS, arylpolyene	Kedarcidin (13%)	*M. chaiyaphumensis, M. humi*
11	Terpene	Nocathiacin (4%)	All but *M. chaiyaphumensis, M. chersina*
12	Type II PKS	Formicamycins A-M (13%)	All
13	NRPS	Azicemicin B (13%)	*M. aurantiaca, M. humi, M. marina*
14	Siderophore	Desferrioxamine E (100%)	All but *M. aurantiaca, M. chalcea*
15	Oligosaccharide, terpene, lanthipeptide	Lobosamide A/B/C (13%)	M. aurantiaca, *M. chalcea, M. humi, M. marina*

16	NRPS, type II PKS	Feglymycin (63%)	None
17	Type II PKS, ectoine, NRPS, type I PKS, other	Kosinostatin (77%)	None
18	Bacteriocin	Lymphostin (33%)	All
19	Terpene	None	All

[a] Percentage values represent sequence identity between the closest BGC relative with strain 28ISP2-46^{T}.

References

1. Ma, H.M.; Zhou, Q.; Tang, Y.M.; Zhang, Z.; Chen, Y.S.; He, H.Y.; Pan, H.X.; Tang, M.C.; Gao, J.F.; Zhao, S.Y., et al. Unconventional Origin and Hybrid System for Construction of Pyrrolopyrrole Moiety in Kosinostatin Biosynthesis. *Chem Biol* **2013**, *20*, 796-805, doi:10.1016/j.chembiol.2013.04.013.
2. Duthie, E.S.; Lorenz, L.L. Staphylococcal Coagulase: Mode of Action and Antigenicity. *Microbiology* **1952**, *6*, 95-107, doi:https://doi.org/10.1099/00221287-6-1-2-95.
3. Hiramatsu, K.; Hanaki, H.; Ino, T.; Yabuta, K.; Oguri, T.; Tenover, F.C. Methicillin-resistant Staphylococcus aureus clinical strain with reduced vancomycin susceptibility. *Journal of Antimicrobial Chemotherapy* **1997**, *40*, 135-136, doi:10.1093/jac/40.1.135.
4. Holloway, B.W. Genetic Recombination in Pseudomonas aeruginosa. *Microbiology* **1955**, *13*, 572-581, doi:https://doi.org/10.1099/00221287-13-3-572.
5. Datsenko, K.A.; Wanner, B.L. One-step inactivation of chromosomal genes in Escherichia coli K-12 using PCR products. *Proc Natl Acad Sci U S A* **2000**, *97*, 6640-6645, doi:10.1073/pnas.120163297.
6. Snijders, E.P.G., W. R. O. Untersuchungen über das Scleroma respiratorium (Sklerom). I. Mitteilung: das Sklerom in Niederländisch-Indien, nebst einigen Bemerkungen über die Epidemiologie dieser Krankheit. *Zentralblatt fur Bakteriologie, Parasitenkunde, Infektionskrankheiten und Hygiene. Erste Abteilung: Originale* **1934**, *133*, 343-348.
7. HUGH, R.; REESE, R. Designation of the type strain for Bacterium anitratum Schaub and Hauber 1948. *International Journal of Systematic and Evolutionary Microbiology* **1967**, *17*, 245-254, doi:https://doi.org/10.1099/00207713-17-3-245.
8. Baumann, P.; Doudoroff, M.; Stanier, R.Y. A study of the Moraxella group. II. Oxidative-negative species (genus Acinetobacter). *J Bacteriol* **1968**, *95*, 1520-1541.
9. Liu, P.V.; Matsumoto, H.; Kusama, H.; Bergan, T. Survey of Heat-Stable, Major Somatic Antigens of Pseudomonas aeruginosa†. *International Journal of Systematic and Evolutionary Microbiology* **1983**, *33*, 256-264, doi:https://doi.org/10.1099/00207713-33-2-256.
10. Horsburgh, M.J.; Aish, J.L.; White, I.J.; Shaw, L.; Lithgow, J.K.; Foster, S.J. σ^B Modulates Virulence Determinant Expression and Stress Resistance: Characterization of a Functional rsbU Strain Derived from Staphylococcus aureus 8325-4. *J Bacteriol* **2002**, *184*, 5457-5467, doi:10.1128/jb.184.19.5457-5467.2002.
